# Acute and post-acute neurobehavioral responses to lysergic acid diethylamide in healthy subjects: a randomized controlled study

**DOI:** 10.1038/s41386-026-02454-7

**Published:** 2026-06-18

**Authors:** Abigail E. Calder, Vincent J. Diehl, Morten P. Lietz, Parsa Yousefi, Nicole Friedli, Fabio Coviello, Antonin Rouaud, Kristian Beichmann, Anne Eckert, Gregor Hasler

**Affiliations:** 1 Molecular Psychiatry Lab, Faculty of Science and Medicine, University of Fribourg, Villars-sur-Glâne, Switzerland; 2 Fribourg Mental Health Network, Villars-sur-Glâne, Switzerland; 3 Lake Lucerne Institute, Vitznau, Switzerland; 4 Neurobiology Laboratory for Brain Aging and Mental Health, Psychiatric University Hospital, University of Basel, Basel, Switzerland

**Keywords:** Long-term potentiation, Pharmacology, Neurophysiology, Learning and memory, Neurotrophic factors

## Abstract

Preclinical studies suggest that lysergic acid diethylamide (LSD) may induce lasting changes in brain function and learning ability, but evidence in humans is uncertain. Motor learning, in particular, has clinical relevance but has not been investigated in human studies of psychedelics. Forty-five healthy subjects (24 women) participated in this randomized crossover trial comparing 100 µg LSD with a placebo. For up to one week after dosing, we investigated LSD’s post-acute neurophysiological effects using auditory tetanization with electroencephalography (EEG), paired associative stimulation (PAS) with transcranial magnetic stimulation (TMS), peripheral levels of brain-derived neurotrophic factor (BDNF). Additionally, online and offline motor learning were assessed one day after dosing with a sequence typing task. Questionnaires assessed perceived stress and cognitive flexibility one week after dosing. We found that offline motor learning significantly improved the day after LSD. One week after LSD, perceived stress was reduced and aspects of cognitive flexibility were increased. EEG data showed that LSD acutely decreased amplitudes of N1 and P2 auditory event-related potentials and still modulated P2 one week later. Motor-evoked potentials measured with TMS showed increased amplitude and faster latency under LSD. LSD did not alter BDNF levels. Our findings encourage future studies on LSD and learning and additionally highlight important challenges in the measurement of long-term potentiation in humans. The observed acute and lasting changes in neural signals provide insight into LSD’s effects on the auditory and motor systems.

## Introduction

Lysergic acid diethylamide (LSD) is a potent serotonergic psychedelic that has re-emerged as a promising compound in the treatment of psychiatric disorders, including major depressive disorder, anxiety disorders, and substance use disorders [1–4]. Notably, a single administration has been associated with therapeutic effects that can persist for months, suggesting that LSD may induce lasting changes in brain function [5–7].

Preclinical research provides important support for this idea. LSD and similar serotonergic psychedelics are thought to produce sustained alterations in neuronal activity, synaptic signaling and functional connectivity which persist beyond the acute drug state and may be driven by changes in dendritic growth, synaptic plasticity, and neurotrophin expression [8, 9]. Behaviorally, LSD is thought to open a critical learning period in which learning is more efficient for at least a few days after dosing [10]. Preclinical studies suggest that serotonergic psychedelics may affect both relatively simple forms of learning, such as classical and operant conditioning [11, 12] and fear extinction [13], but also more complex processes like social reward learning, possibly for at least several days after exposure [10, 11]. These findings indicate that LSD may induce enduring functional changes in the brain that could underlie its long-term behavioral and clinical effects.

However, evidence in humans remains relatively limited, particularly with regard to post-acute effects. While acute subjective and neural effects of LSD are relatively well described, less is known about how brain function and behavior evolve in the days following administration. Specifically, lasting effects on motor learning and perceptual processing have not been systematically investigated after LSD exposure in controlled experimental settings.

In the present study, we examined potential post-acute effects of LSD using a multimodal approach. Neurophysiological measures included electroencephalography (EEG) with sensory tetanization (ST) in the auditory domain to assess effects on sensory processing, as well as transcranial magnetic stimulation (TMS) with paired associative stimulation (PAS) to investigate changes in motor system excitability; both ST and PAS were also probed as measures of functional neuroplasticity [14, 15]. In addition, we assessed motor learning performance using a sequence typing task capturing both online learning during active practice and offline consolidation following practice [16]. Self-report measures included questionnaires on perceived stress and cognitive flexibility. Together, these approaches allowed us to characterize acute and longer-term effects on perceptual processing, motor learning, and psychological functioning for up to one week after a single dose of LSD in healthy subjects.

## Methods and materials

### Study design and participants

This study used a randomized, double-blind crossover design. Forty-five healthy participants between the ages of 21 and 55 received one dose of 100 µg LSD and one inactive placebo (Apotheke Dr. Hysek AG, Biel, Switzerland). All participants provided written informed consent and were compensated. The study was conducted in accordance with the Declaration of Helsinki, approved by the Bern Cantonal Ethical Committee (BASEC-No. 2021-01322) and registered on ClinicalTrials.gov (NCT05177419). See supplement for details on inclusion criteria and drugs.

### Study procedures

**Fig. 1:**
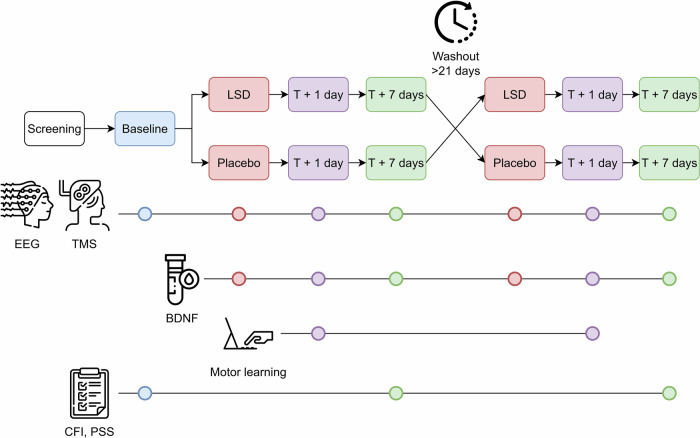
Study procedures at eight study appointments in a crossover design. Colored dots represent the experiments conducted at each appointment. BDNF brain-derived neurotrophic factor, CFI Cognitive Flexibility Inventory, EEG electroencephalography, PSS Perceived Stress Scale, T time of dosing, TMS transcranial magnetic stimulation.

Participants completed a screening visit and seven experimental sessions including the two dosing days (Fig. 1; see supplement for details). Dosing sessions were conducted in a quiet, comfortable room with one or two investigators present at all times (Supplementary Fig. S1). A washout period of >4 weeks was required between the LSD and placebo appointments. Baseline measures of EEG and TMS were taken at the baseline visit and repeated six and seven hours after drug administration, respectively, as well as the next day and 6–8 days later (mean: 7.01). Motor learning was completed the day after dosing. Questionnaires were completed at baseline and one week after dosing. Experiments were always conducted in the afternoon in identical order and participants reported on hours of sleep and exercise in the past 24 h.

### EEG procedures

Auditory ST (Supplementary Fig. S2) was used to assess acute and post-acute changes in auditory ERPs in response to LSD. In addition to information on ERPs, ST was developed as a translational measure of long-term potentiation (LTP) in humans [17]. It thus served as an attempt to index LTP-like changes over frontocentral electrodes following a protocol used in previous studies [17, 18].

EEG recordings were conducted using 32 actively shielded wet electrodes (actiCAP slim EEG system) in 10-20 system locations with an electrode cap and a LiveAmp amplifier (Brain Products GmbH, Germany). Signals were recorded at 1000 Hz and impedances were kept below 25kΩ as recommended by the manufacturer for actively shielded electrodes. Participants were instructed to sit still and attend to a series of identical tones (1000 Hz with Hanning window at edges, 50 ms duration, 60 dB) over in-ear headphones (ER-3C, Etymotic Research, IL, USA) with eyes open. In the first block, single tones were played at intervals of 1800–2600 ms for 4.5 min. The tetanization block was played after a one-minute break and consisted of tones played at 13 Hz for two minutes. Finally, after a two-minute break, the first block was repeated three more times at 15-min intervals.

Pre-processing was conducted offline using EEGLAB (Version 2024.2) [19]. Data were downsampled to 250 Hz and bandpass filtered between 1 and 30 Hz. Epochs began 200 ms before stimulus onset and ended 600 ms after onset. A baseline correction was applied using the 200 ms before stimulus onset. Bad channels were interpolated and large artefacts were removed, then independent component analysis was used to remove eye movement artefacts. Smaller artefacts were flagged using a custom script and approved for removal by manual inspection.

Similar to previous auditory ST studies [18], we analyzed the amplitude of auditory N1 (greatest negative peak 50–150 ms after stimulus) and P2 (greatest positive peak 150–250 ms after stimulus) ERP components averaged over frontocentral electrodes (Cz, Fz, FC1, and FC2), both before and after tetanization. This choice of electrodes is consistent with previous studies [17, 18], and it also reflects the region at which both components showed the strongest signal in our data (Supplementary Fig. S3). Participants who showed any increase in ERP amplitude after ST were classified as “responders” in calculations of responder rates, consistent with previous studies [18]. Finally, we attempted to replicate a previous exploratory analysis from a study using ST [20], which demonstrated a trend-level increase in evoked theta power two weeks after psilocybin treatment (see Supplemental Methods).

### TMS procedures

The TMS-based PAS method was used to investigate both motor cortical excitability and LTP-like changes in the motor domain during and after LSD exposure [21]. PAS was conducted using a MagPro X100 (Tonica Elektronik A/S, Denmark) coupled to a Dantec Keypoint electrical stimulator (Alpine Biomed ApS, Skovlunde, Denmark) and adhesive electrodes for electromyography of the right abductor pollicis brevis (APB). In preparation for TMS, participants were fitted with electrodes for measuring motor-evoked potentials (MEPs) over the abductor pollicis brevis (APB) of the right hand using the belly-tendon configuration [22]. Participants wore a reusable cap on which the approximate location of the motor hotspot was marked using the C3 point on the 10/20 system. The TMS coil was placed over this hotspot at an angle of 45 degrees to the midline, and the correct hotspot was identified by determining the point at which a TMS pulse of 70% maximum strength evoked the highest amplitude in the right APB. Subsequently, pulse strength was calibrated so that a single pulse produced an MEP with a peak-to-peak amplitude of ~1 mV in the relaxed APB. After calibration at the baseline visit, pulse strength and hotspot location were recorded and kept consistent for each participant throughout the study.

We used a prototypical PAS procedure as described in previous studies (Supplementary Fig. S2) [23]. Monophasic TMS pulses were always delivered at a rate of 0.1 Hz. First, baseline MEP amplitude in the right APB was determined using a train of 20 TMS pulses. Next, paired stimulation was applied using 180 TMS pulses paired with peripheral electrical stimulation of the right median nerve at 300% of the perceptual threshold, a strength that is noticeable but painless. Median nerve stimulation was delivered with an interstimulus interval of 25 ms before the TMS pulse so that both arrived at motor cortical synapses simultaneously, which is presumably required for induction of Hebbian LTP [24]. Participants were asked to keep the right hand relaxed and silently count the number of pulses in order to keep their attention on the stimuli. Finally, the baseline train of TMS pulses was repeated three times at intervals of 15 minutes (2, 17 and 32 min after the end of the paired pulses) in order to measure changes in MEP amplitude after paired stimulation.

Prior to analysis, MEP peak and latency detection were performed with custom R scripts and confirmed by manual inspection. MEPs with a peak-to-peak amplitude of <50 µV were rejected [25]. Latency was defined as the earliest timepoint at which an MEP deviated from baseline motor activity by at least 25 µV. Participants who showed any increase in MEP amplitude after PAS were classified as “responders” in calculations of responder rates, consistent with previous studies [26]. When displaying grand average MEP waveforms, positive MEP peaks were aligned to the median peak latency of the given condition and visit. This ensured that grand averages preserved true peak amplitudes without flattening due to latency variability.

### BDNF

To assess possible longer-term effects of LSD on neurotrophin expression [27], BDNF was measured in blood plasma and serum before drug intake, as well as eight hours, one day and one week later. Because previous studies have typically used only one blood fraction [28, 29], we chose to measure BDNF in both plasma and serum in order to compare their sensitivity to LSD. Plasma samples were collected with EDTA tubes and serum samples were collected using rapid serum tubes. Serum samples were allowed to clot for five minutes according to manufacturer instructions. Samples were immediately centrifuged for 10 min at 3000 RPM, after which serum and plasma were extracted and frozen at −80° until analysis. BDNF levels were measured using the Biosensis Mature BDNF Rapid ELISA Kit (Thebarton, Australia).

### Motor learning

**Fig. 2:**
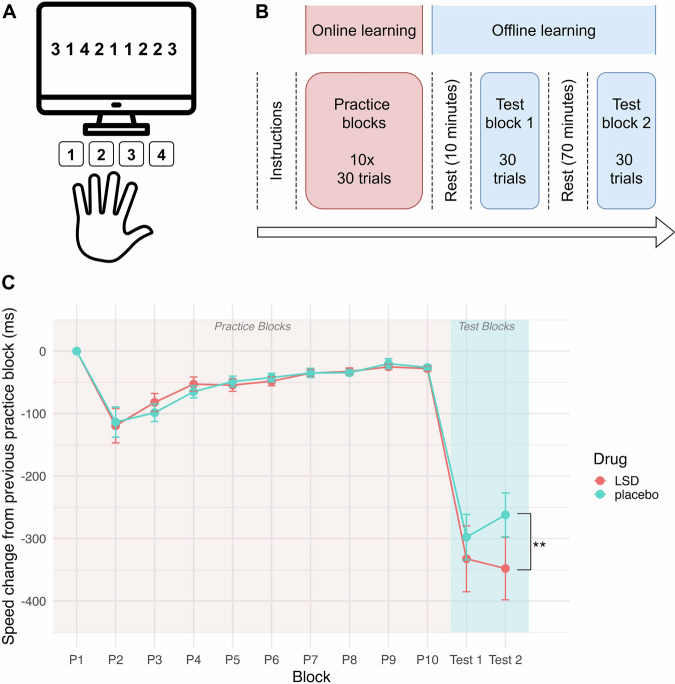
Procedures and results of the sequence typing task used to assess online and offline motor learning. **A** Participants placed their left hand on a standard computer keyboard and were instructed to type a 9-digit key sequence as quickly and accurately as possible. **B** Participants practiced the sequence repeatedly during ten practice blocks consisting of 30 trials each, as well as in two test blocks after resting. During the 10-min break before the first test block, they rested while sitting with eyes closed. During the remaining 70 min before the final test block, participants completed other unrelated experiments (passive EEG recordings, questionnaires). **C** Improvement in typing speed in the motor learning task across ten consecutive practice blocks (P1–P10; online learning) and two test blocks (offline learning) completed after resting for 10 and 80 min. Participants showed significantly more speed improvement in the test blocks one day after LSD compared to placebo, mostly driven by a difference in improvement after 80 min of rest. Error bars show SEM. *N* = 42. **p < 0.01.

One day after drug administration, participants completed a sequence typing task developed based on previous studies (Fig. 2A, B) [16]. It assessed both online learning during active practice of motor movements and offline learning improvements after rest [30–32]. Participants were instructed to type a 9-digit number sequence (314211223 or 322112413, counterbalanced across drug order) as quickly and accurately as possible using their left (non-dominant) hand. We ensured comparable difficulty between the two sequences by including the same number of taps for each finger, as well as the same set of finger transitions, in both sequences. The task consisted of 10 practice blocks (online learning) and two test blocks after 10 and 80 min of rest (offline learning). Audiovisual feedback on performance was given after each trial and block, and participants were encouraged to beat their high scores from the previous block. Learning in each block was operationalized as the change in average typing speed on correct trials since the previous practice block.

### Questionnaires

Acute drug effects were assessed using verbal hourly ratings [1, 33], as well as the 5-Dimensional Altered State of Consciousness Questionnaire (5D-ASC) and the Mystical Experience Questionnaire (MEQ) [34–36]. Side effects were assessed using the Swiss Psychedelic Side Effects Inventory (SPSI) [37]. Cognitive flexibility was assessed with the Cognitive Flexibility Inventory (CFI) and perceived psychological stress was measured using the Perceived Stress Scale (PSS) [38, 39]. See supplement for details.

### Data analysis

Statistical analyses were conducted using R (Version 4.5.0) [40]. Effects of LSD were analyzed using linear mixed effects models with fixed effects for drug, visit, and treatment order and random effects for individual participants (*lme4* package, version 1.1.37 [41]). Additionally, models assessing hourly ratings of acute drug effects included drug x hour interactions as fixed effects. Models assessing effects of PAS, ST, BDNF, and motor learning included participants’ reported number of hours of exercise and sleep in the past 24 h as covariates [42]. When applicable, baseline values were included as covariates in the models. The two models assessing N1 and P2 auditory ERP amplitudes included fixed effects for tetanus condition (pre- or post-tetanus), and all post-tetanus timepoints were included individually without averaging or other transformations. These models also included three-way interaction effects between drug, visit and tetanus condition. The two models assessing TMS amplitude and latency included fixed effects for PAS condition (pre- or post-PAS) and interaction effects in the same manner, and they additionally included a fixed effect for number of pulses counted during PAS. Following the lack of tetanus and PAS effects, the models were re-run without effects for tetanus and PAS conditions. Models assessing motor learning included fixed and effects for learning condition (online or offline) with each block included individually, as well as an interaction effect between drug and learning condition. Robust estimators were used when assumptions of linear mixed effects models were violated (*robustlmm* package, version 3.3-3 [43]). Because there were no significant order effects, all LSD and placebo visits were analyzed together. Post-hoc contrasts at each timepoint (on dosing days, one day later, and one week later) were conducted using estimated marginal means using either pairwise comparisons or linear trends (*emmeans* package, version 1.11.1 [44]). *P* values were adjusted using the multivariate *t* method to control the familywise error rate. Sensitivity analyses were used to test whether results were driven by outliers or influential cases. Correlations were calculated using Spearman’s correlation coefficient with FDR correction for multiple comparisons.

## Results

### Sample characteristics

Forty-five volunteers were included in the study, and data from 43 (24 women, mean age = 30.4) were analyzed (CONSORT [45] diagram in Supplementary Fig. S4). Demographics are shown in Supplementary Table S1.

### Subjective drug effects and side effects

LSD significantly increased hourly ratings of most drug effects, as well as scores on the 5D-ASC and MEQ (Supplementary Fig. S5, Supplementary Table S2). Most participants reported positive effects of LSD on well-being (Supplementary Table S3). Regarding blinding, all participants correctly identified the 100 µg LSD dose. Blinding of the placebo as low-dose LSD was successful in 79% of participants (mean guess = 14 µg, SD = 11.3). LSD increased the number of acute and post-acute side effects for up to 24 h, though they were generally mild and transient (Supplementary Tables S4, S5, Supplementary Fig. S6). There was one possibly treatment-related serious adverse event that resolved within the study timeframe (see supplement for a detailed discussion of this and other side effects).

### EEG outcomes

We first investigated whether ST caused an LTP-like potentiation of ERPs. Contrary to expectations, there was no main effect of ST on amplitude of N1 (β = 0.05, SE = 0.13, *p* = 0.70) or P2 (β = 0.15, SE = 0.18, *p* = 0.41) (Supplementary Table S6, Supplementary Fig. S7). The ST responder rate was 43% for N1 and 53% for P2. Planned contrasts by visit revealed that one week after treatment, P2 amplitude significantly decreased after ST in the LSD condition only (β = −0.47, SE = 0.17, *p* < 0.05). The corresponding drug x ST interaction was initially significant (β = −0.51, SE = 0.25, unadjusted *p* = 0.04) but did not survive correction (adjusted *p* = 0.14). There were no other significant drug x ST or visit x ST interactions. To further investigate the post-tetanus reduction in P2 at one week, we analyzed change in P2 amplitudes over all four recording timepoints within that EEG session (one pre-ST, three post-ST). P2 amplitude significantly decreased in the LSD condition compared to the placebo condition (β = −0.22, SE = 0.10, *p* < 0.05) with a steady linear trend across all four timepoints (Supplementary Fig. S8); this is not consistent with the expected effects of ST.

**Fig. 3:**
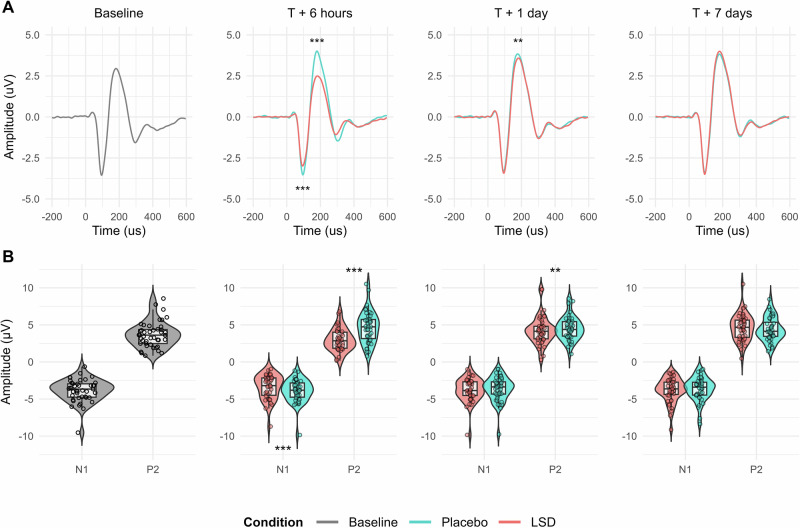
Event-related potentials (ERPs) at baseline and at three timepoints after LSD and placebo administration. **A** ERP waveforms showing both N1 and P2 components at each timepoint. **B** ERP amplitudes at each timepoint. On dosing days, N1 and P2 amplitudes were significantly decreased in the LSD condition. P2 was still decreased in the LSD condition the following day. Significance stars show differences in amplitude between conditions. T time of drug intake. *N* = 43. ***p < 0.001, **p < 0.01.

Regardless of ST, LSD showed main effects on ERP amplitudes (Fig. 3, Supplementary Fig. S9, Supplementary Table S7). Models analyzing all N1 and P2 ERPs together for each visit showed that LSD acutely and significantly reduced N1 (β = 0.5, SE = 0.08, *p* < 0.001) and P2 (β = −1.33, SE = 0.11, *p* < 0.001) amplitudes. P2 amplitude was still significantly reduced one day after LSD (β = −0.32, SE = 0.11, *p* < 0.01), which was the only significant post-acute effect. There was no effect of LSD on evoked theta power at any timepoint (Supplementary Table S8).

### TMS outcomes

We first analyzed whether PAS induced LTP-like effects. There was neither a main effect of PAS on MEP amplitude (β = 7.4, SE = 43.9, *p* = 0.87) nor any significant PAS x drug interaction for any visit (Supplementary Table S9). The responder rate for PAS was 53.4%. We therefore analyzed all MEPs for each visit together to assess LSD’s impact on corticospinal excitability only.

**Fig. 4:**
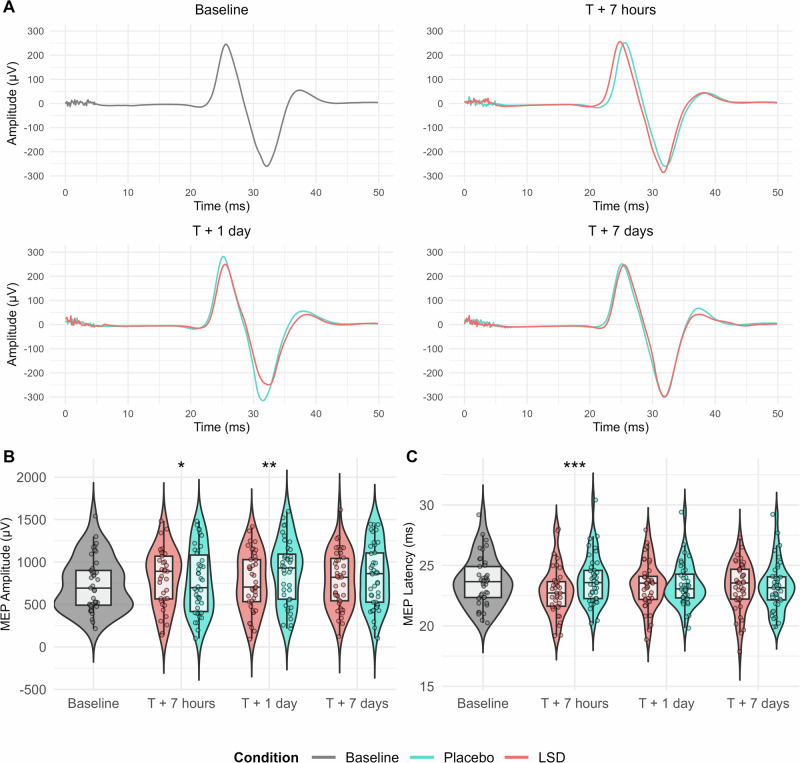
Motor-evoked potentials (MEPs) at baseline and three timepoints after LSD and placebo administration. **A** MEP waveforms at each timepoint. **B** Peak-to-peak MEP amplitudes at each timepoint. On dosing days, MEP amplitudes were significantly larger in the LSD condition. The next day, MEP amplitudes were significantly decreased in the LSD condition. **C** MEP latencies at each timepoint. On dosing days, MEP latencies were significantly shorter in the LSD condition. T time of drug intake. *N* = 42. ***p <0.001, **p <0.01, *p <0.05.

MEP waveforms are shown in Fig. 4A. There was a significant LSD effect on MEP amplitude (β = 64.4, SE = 43.9, *p* < 0.01). Post-hoc tests revealed that MEP amplitude was significantly increased in the LSD condition on dosing days (β = 64.4, SE = 26.6, *p* < 0.05) and significantly decreased in the LSD condition one day after dosing (β = −80.8, SE = 26.1, *p* < 0.01), compared to placebo (Fig. 4B; Supplementary Fig. S10, Supplementary Table S10). Additionally, there was a main effect of LSD on MEP latency (β = −775.2, SE = 119.8, *p* < 0.001). Post-hoc tests showed that this effect was driven by dosing days, on which the LSD condition showed significantly decreased MEP latency (β = −775.2, SE = 119.8, *p* < 0.001) (Fig. 4C, Supplementary Fig. S10). During PAS on dosing days, participants in the LSD condition counted a mean of 163 TMS pulses compared to 177 (out of 180) in the placebo group; this effect approached significance (β = 4.69, SE = 2.67, *p* = 0.08). The number of pulses counted did not differ between groups one day (β = −0.71, SE = 2.63, *p* = 0.79) or one week after dosing (β = −1.58, SE = 2.58, *p* = 54).

### BDNF

There was no effect of LSD on BDNF in either serum (β = 0.49, *t* = 0.41, *p* = 0.69) or plasma (β = 0.26, *t* = 1.6, *p* = 0.11) at any timepoint (Supplementary Table S11).

### Motor learning

On the motor learning task, mixed effects models showed a significant main effect of condition (β = −216.2, SE = 7.4, *p* < 0.001), indicating that participants’ typing speed improved in the test blocks (offline learning) relative to practice blocks (online learning). There was also a significant drug x condition interaction (β = 24.4, SE = 10.5, *p* < 0.05). Post-hoc tests showed that the LSD condition showed greater offline speed improvements than the placebo condition (β = −22.32, SE = 9.7, *p* < 0.05; Fig. 2C, Supplementary Table S12). This was primarily driven by the second test block completed 80 minutes after the end of practice (β = −37.0, SE = 12.6, *p* < 0.01). In the second test block, the LSD condition was 348 ms faster and the placebo condition was 262 ms faster than in the final practice block, corresponding to a 32.8% greater improvement in the LSD condition. LSD did not affect speed improvement during practice (β = −2.1, SE = 4.8, *p* = 0.66), initial speed in the first practice block (β = 38.2, SE = 81.0, *p* = 0.64) or accuracy during online (β = 0.8, SE = 0.6, *p* = 0.16) or offline learning (β = 1.1, SE = 1.2, *p* = 0.36), nor were there block-level effects on accuracy. Finally, neither motor learning nor questionnaire scores correlated with neurophysiological outcomes (Supplementary Table S13).

### Cognitive flexibility and perceived stress

**Fig. 5:**
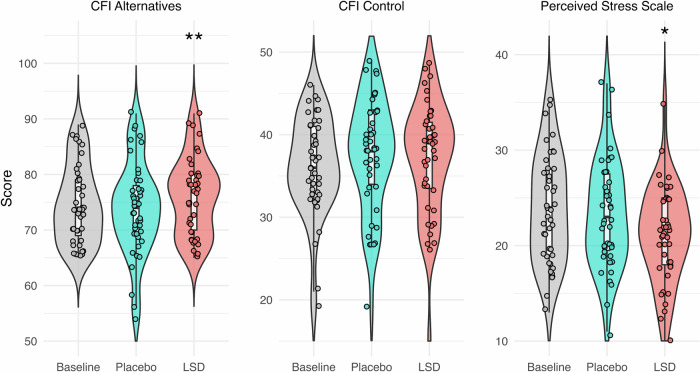
Scores on the two subscales of the Cognitive Flexibility Inventory (CFI), as well as the Perceived Stress Scale (PSS), at baseline and one week after drug administration. On the CFI, possible scores range from 13 to 91 for Alternatives and 7 to 49 for Control. Possible PSS scores range from 10 to 50. Statistical tests reflect contrasts between LSD and placebo with baseline scores as a covariate. Error bars show SEM. *N* = 43. *p < 0.05, **p < 0.01.

One week after dosing, LSD significantly increased ratings on the *Alternatives* subscale of the CFI (β = 1.92, SE = 0.63, *p* < 0.01), but not *Control* (β = −0.53, SE = 0.67, *p* = 0.42; Fig. 5, Supplementary Fig. S11, Supplementary Table S14). LSD also significantly reduced PSS scores (β = −1.94, SE = 0.91, *p* < 0.05; Fig. 5, Supplementary Fig. S11, Supplementary Table S14).

## Discussion

To our knowledge, this randomized controlled crossover trial in healthy subjects is the first to demonstrate improvements in motor learning after a psychedelic drug, as well as the first to report LSD’s acute and post-acute effects on MEPs and auditory ERPs at a fully psychedelic dose. LSD also led to improvements in perceived stress and aspects of cognitive flexibility. Like previous studies, LSD induced mostly positive acute and lasting subjective effects, as well as transient side effects [1, 33, 36, 46, 47].

Acutely, LSD reduced the amplitude of the N1 and P2 components of auditory ERPs, and the reduction in P2 lingered one day later. N1 reflects early, bottom-up auditory processing and properties of the sensory stimulus (e.g. volume) [48]. P2 is thought to reflect early attentional orientation and is sensitive to changes in stimulus familiarity and novelty [49, 50]. Our results align with previous research reporting that serotonergic psychedelics acutely reduce the amplitude of various sensory ERPs. Auditory N1 is attenuated by psilocybin, as is auditory P3, which is associated with higher-order cognitive processing [51, 52]. LSD and psilocybin also both reduce the amplitude of certain visual ERPs, including both early perceptual ERPs (e.g. N170) and later cognitive ones (e.g. P3) [53, 54]. These ERP reductions likely depend on 5-HT2A receptor activation [54], in line with the observation that serotonergic tone mediates N1 amplitude after auditory stimuli [52]. At the network level, ERP modulation may reflect LSD-induced desynchronization of neural activity associated with disruption of early sensory processing and attentional allocation [51, 53], possibly due to competition for neural resources between external stimuli and internally generated stimuli (i.e., pseudo-hallucinations) [55]. LSD may acutely impair attentional control [56], and the trend toward fewer counted TMS pulses on dosing days in the LSD group also indicates acute effects on attentional control in this sample, which could have been reflected in sensory ERPs, particularly P2.

Tetanization did not change auditory ERP amplitudes in this sample. Three other recent studies have also attempted to use ST to measure functional LTP-like changes after serotonergic psychedelics. Like the current study, all reported post-acute changes in electrophysiological signals, but without the expected potentiation of ERP amplitudes [20, 57, 58]. A study of high dose psilocybin reported a trend-level increase in evoked theta power during auditory ST, which we did not observe in this sample [20]. A study of low dose LSD (14 ×10 µg over 6 weeks) reported changes in laminar connectivity in the primary visual cortex two days after the final dose [58]. Interestingly, a second low-dose LSD study (4 ×15 µg over two weeks) reported greater amplitude of the auditory P3a component during a mismatch negativity paradigm one week after the final dose [57]. This was interpreted as an effect of LSD on pre-attentive processing and novelty detection. In the current study, the LSD condition showed a specific, linear decrease in P2 amplitude over the 45-min recording session one week after dosing. P2 amplitude is typically stable within a recording session but can decrease when a stimulus is presented repeatedly, a process called habituation [59]. Speculatively, a gradual reduction in P2 amplitude could reflect a delayed effect of LSD on attention or habituation to a decreasingly novel stimulus. However, this result is difficult to interpret without any concurrent measures of other physiological or subjective variables during EEG (e.g. measures of physical arousal or attention). Overall, the lasting effects on electrophysiological signals seen in this and other studies are exploratory, difficult to interpret and in need of replication.

During TMS, LSD acutely increased peak-to-peak MEP amplitude and decreased latency, compared to placebo. Ketamine, SSRIs, and psychostimulants also acutely enhance MEP amplitude [60]. Faster, larger MEPs could reflect increased corticospinal excitability, peripheral alterations in muscle tension, or both. It is possible that LSD enhances corticospinal excitability via enhanced glutamatergic neurotransmission in motor cortical neurons expressing 5-HT2ARs [61–63]. Decreased MEP amplitude the day after LSD compared to placebo could conceivably be related to fatigue [64], which was greater in the LSD condition. On the other hand, LSD commonly causes muscle tension and shaking during peak drug effects [37], including in this sample, which may also depend on 5-HT2A receptor-dependent glutamate receptor activation [65, 66]. Greater muscle contraction increases MEP amplitudes and decreases latency [61]. Though participants were instructed to relax the right hand and visual inspection of MEP curves does not suggest greater pre-MEP muscle contraction in the LSD condition eight hours after dosing, we did not quantify muscle contraction prior to TMS pulses and thus cannot tell whether MEP effects are of peripheral or central origin. Further studies accounting for LSD effects on peripheral muscle tension are needed to clarify whether LSD-induced changes in MEP amplitude reflect central changes in corticospinal excitability or peripheral muscular effects, as well as whether these effects could have clinical potential for improving motor function in neurorehabilitation or other clinical settings.

We found no LSD effect on plasma or serum BDNF, consistent with a recent meta-analysis which found no effect of serotonergic psychedelics or other *psychoplastogens* [67] on peripheral BDNF in humans [27]. Peripheral BDNF is attractive as a biomarker because it can be measured non-invasively with a simple blood draw, and it may correlate with brain BDNF under some circumstances [68]. However, blood BDNF mostly does not originate from the brain and may not be sensitive enough to detect the rapid upregulation of brain BDNF seen in preclinical studies, assuming it happens in humans at all [69, 70].

Relatedly, there is a need for reliable translational measures of psychedelic-induced neuroplasticity, which is consistently reported in preclinical studies but challenging to measure in humans [9, 71]. The fact that neither ST nor PAS induced LTP-like changes in this or other recent studies with psychedelics is worth critical consideration. Both ST and PAS were developed as translational measures of LTP mimicking the effects of rapid electrical stimulation (*tetanization*) of neurons in vitro [21, 72]. However, both are also limited by high response variability, the sources of which are not well understood [73–76]. Responder rates for both paradigms hover around 50–60% [18, 77], which may resemble chance levels and is much less reliable than the electrophysiological induction of LTP in vitro that ST and PAS were developed to mimic [73]. Our sample had similar responder rates and variability, thus suffering from some of the same problems noted by other authors. At the sample level, while some studies using ST or PAS observe the expected overall increases in signal amplitude, others observe no effect or even decreases, like in our sample [18, 77]. Unlike early studies of ST [17, 78], a recent meta-analysis has suggested that ST does not reliably modulate ERP amplitude in either the auditory or visual domain [79]. Findings on PAS are more mixed. While one quantitative review reported overall significant effects of PAS with exactly the parameters used in this study [80], a more recent pooled analysis found no effect and suggested that MEP signal potentiation may be a statistical artefact driven by positively skewed, highly variable data, which is sensitive to outliers, a critique which could also apply to ERPs in ST [77].

Given these limitations, future studies using ST or PAS may benefit from improving their reliability. Though there is no clear consensus on how this may be achieved, previous research suggests using larger sample sizes to improve statistical power [18], only including responders in studies [42, 75], optimizing attention and alertness during experiments [73], and calculating custom interstimulus intervals for PAS based on individual nerve conduction speed [24]. For ST, one study has suggested that noise bursts may induce more stable LTP-like changes than pure tones [81]. Furthermore, while neither auditory or visual ST appears to be particularly reliable [79], some have suggested that the visual paradigm is superior [18]. However, we would also acknowledge that the translational validity of ST and PAS may be fundamentally questionable. Both were designed to mimic the effects of electrophysiological stimulation of neurons in vitro, which reliably induces LTP [21, 72]. Without reliable effects in humans, the assumption that paired or rapid sensory stimulation of the living nervous system engages the same mechanisms as direct electrophysiological stimulation of a single neuron could be a flawed translational leap. Other translatable markers of psychedelic-induced neuroplasticity may be more promising, for example imaging of synaptic proteins using positron emission tomography [71, 82].

One day after dosing, participants in the LSD condition showed selective improvements in motor consolidation after rest (offline learning), though not during active practice (online learning). This result was specific to improvements in speed. There was no change in accuracy, suggesting that the effect on speed does not simply reflect a speed-accuracy trade-off [83]. Possible explanations for this include post-acute enhancement of functional plasticity, but also post-acute effects on motivation, attention, and/or arousal. Concerning the former, periods of waking rest after learning facilitate memory consolidation, leading to performance improvements without additional practice [30, 32, 84]. These offline learning gains are associated with LTP and changes in brain microstructure thought to reflect early synaptic consolidation and stabilization [84–88]. Preclinical research suggests that LSD and other psychedelics may broadly enhance synaptic plasticity in the neocortex and hippocampus [9], important regions for motor skill consolidation [84, 86], as well as improve performance on diverse learning paradigms, including classical and operant conditioning [11, 12], fear extinction [13], and social reward learning [10, 11]. In humans, one previous study of 50 µg LSD also found improvements in visuospatial learning and memory consolidation the day after dosing [89].

On the other hand, offline learning improvements may also be explained by lingering LSD effects on motivation, attention, and/or arousal one day after dosing. Acutely, LSD increases reward-related brain activity and sensitivity to reinforcement learning [90, 91]. Should this effect persist into the next day, participants may be more motivated during learning tasks and thus perform better [92]. Complicating matters, participants in the LSD condition reported greater fatigue and low mood on the SPSI one day after dosing. They also showed reduced MEP amplitude after TMS pulses compared to the placebo condition, which upon visual inspection seemed to be driven by an increase in the placebo condition compared to baseline. Fatigue and comparatively low arousal would be expected to impair motor learning [93, 94]. It is unclear how the potentially beneficial effects of reward sensitivity and potentially negative effects of fatigue might combine to affect learning. The lack of correlation between motor learning and any of the neurophysiological variables also does not support clear interpretations. On balance, it remains unclear whether the selective offline learning improvement one day after LSD reflects altered functional plasticity, lingering effects on arousal, attention, and motivation, or a mix of these variables. Future studies of psychedelics and learning should measure the effects of motivation and arousal more precisely, as well as investigate whether learning effects are observable at later timepoints, in different populations, or in other learning paradigms.

LSD significantly reduced perceived stress one week after dosing, consistent with other studies reporting positive changes in mood and well-being after psychedelic use [95]. Additionally, LSD increased scores on the *Alternatives* subscale of the CFI one week after dosing. Notably, baseline CFI and PSS scores were close to maximum and minimum values, respectively. People with lower baseline cognitive flexibility or higher subjective stress may have more room to benefit, as implied by previous studies of psychedelics in both clinical and naturalistic contexts, which found positive effects on cognitive flexibility and stress-related outcomes [96–98]. However, it is important to note that unblinding was high in both this and other studies assessing self-reported positive effects from psychedelics. It is possible that at least some of the observed effects on cognitive flexibility and stress are placebo effects reflecting generally positive expectations of psychedelic drugs [99, 100]. Future studies would benefit from including task-based measures of cognitive flexibility and stress, which are less susceptible to expectancy effects, as well as assessing participants’ expectations about specific drug effects as possible confounders.

### Limitations

This study had several important limitations. The sample size of 43 may have been underpowered for detecting some interaction effects or complex order effects. Because ST and PAS did not potential neural signals, we could not evaluate LSD’s effects on LTP. The effect of LSD on P2 ERPs one week after dosing should be considered exploratory. Additionally, the baseline covariates included in statistical models were not re-assessed at each visit and we did not include plasma drug concentrations. The motor learning task was only given one day after dosing due to time constraints, but future studies would benefit from investigating the effects of LSD on learning at later timepoints, as well as whether learning gains are stable over time. Several findings were difficult to interpret mechanistically without quantification of possible confounders. Specifically, we did not measure possible confounding factors of motivation, attention or arousal during motor learning or EEG acquisition, which could have impacted the results, nor did we quantify peripheral muscle activity prior to TMS pulses. Finally, we attempted to reduce placebo effects by presenting the placebo as a low dose of LSD, but all participants still identified the real LSD dose due to its strong psychoactive effects. Because many participants in trials of psychedelics have positive expectations of how the drug will affect them, this failure of blinding could have strongly influenced self-report and behavioral outcomes [101].

## Conclusions

A single dose of LSD improved motor learning performance the next day, as well as perceived stress and aspects of cognitive flexibility one week later. Motor responses to TMS pulses showed greater amplitude and decreased latency under LSD, possibly reflecting increased corticospinal excitability or peripheral muscle contraction. Auditory N1 and P2 ERPs showed reduced amplitude under LSD, and modulation of P2 lingered for up to one week. This and other studies show that stimulation-based markers of LTP are methodologically challenging, encouraging future studies using alternative markers, for example PET imaging. LSD’s possible effects on learning deserve more detailed study.

## Supplementary information

Supplemental Material

## Data Availability

The data supporting the conclusions in this article are available from the corresponding author upon reasonable request.
