# Supplementary material for: Acute and post-acute neurobehavioral responses to lysergic acid diethylamide in healthy subjects: a randomized controlled study
Source: Neuropsychopharmacology. 2026 Jun 18;51(9):1577–87. doi: 10.1038/s41386-026-02454-7 (PMC13389031; doi:10.1038/s41386-026-02454-7)
Supplement: Supplementary file 1 — Supplemental Material [file 41386_2026_2454_MOESM1_ESM.docx]

**SUPPLEMENT**

**Acute and post-acute neurobehavioral responses to lysergic acid diethylamide in healthy subjects: a randomized controlled study**

*Calder et al. 2026*

# Supplemental Methods

## Participants and inclusion criteria

Participants were recruited via study advertisements and word of mouth. Inclusion criteria were fluency in German, healthy weight range (BMI > 18 and < 29), right-handedness (Edinburgh Handedness Inventory > 60), and normal or corrected to normal hearing and vision. Sexually active women of childbearing age were required to be using effective birth control. Participants were excluded for the following reasons: any history of psychosis, mania, suicidality, or neurological or cardiovascular disease; a first-degree relative with any psychotic symptoms; any psychiatric illness within the past three years; pregnancy and nursing; current use of psychoactive medications; implanted metal or electronic devices; daily or near-daily consumption of alcohol, nicotine, or illicit substances; use of serotonergic psychedelics on more than 10 previous occasions or within the prior 3 months. Participants were required to refrain from illicit substances for the duration of the study, as well as from consuming alcohol and nicotine in the 24 hours before any study appointment.

Determination of sample size

*A priori* power analysis was conducted to estimate the required number of participants for adequate statistical power to detect drug effects on PAS. Effect size was estimated from a previous crossover study of citalopram effects on PAS in healthy subjects [1]. Means and SEMs were extracted from plots using WebPlotDigitizer [2] and used to calculate the observed effect size, Cohen’s *d* = 0.498. At an alpha level of 0.05, we determined that 34 participants would be needed to detect an effect of this size with a power level of 0.80 and 45 would be needed to do so with a power level of 0.90. We chose to recruit 45 participants to ensure adequate power.

## Study drugs and randomization

The use of LSD was approved by the Swiss Federal Office of Public Health. Units of 100µg LSD base (Bözinger Apotheke, Biel, Switzerland) were produced according to good manufacturing practice and administered orally in 1mL ethanol. Placebo solutions were visually identical and contained 1mL ethanol. The manufacturer randomly allocated participants to receive LSD or placebo first and study staff administering doses were blind to the randomization.

## Detailed experimental procedures

**Screening.** Screening consisted of an interview assessing inclusion criteria, including the SCID-5-CV diagnostic interview [3]. It also included preparation for the effects of LSD (see “Preparation and setting for LSD sessions” in the final section of the supplementary materials below).

**Blinding.** At the screening visit, participants were told that they would receive one moderately high dose of 100µg LSD and one lower, unknown dose of LSD (actually the placebo; participants were debriefed at the final visit). This was done in order to make the impact of placebo effects between the two drug conditions as comparable as possible. We chose to use an inactive placebo rather than a small dose of LSD because we could not rule out the possibility that even small doses of LSD have sensory, motor and neuroplastic effects [4].

**Baseline visit.** Baseline measurements were taken in the afternoon of the second study visit. Participants arrived in the early afternoon and first underwent sensory tetanization (ST). Questionnaires were filled out in the breaks and after the EEG recording. Participants then underwent paired associative stimulation (PAS) and, finally, a second preparatory session for the LSD day (see below).

**Measurements on dosing days.** Participants arrived between 8:00 and 9:00 in the morning and were advised to eat a light breakfast and consume no more caffeine than usual. Urine tests confirmed abstinence from illicit drugs and absence of pregnancy. A blood draw was done shortly after arrival to assess baseline BDNF levels prior to drug administration. LSD or placebo was administered orally in a double-blind manner one hour after participants’ arrival. Once per hour for the first eight hours after drug administration, participants verbally rated the intensity of overall drug effects, pleasant effects, unpleasant effects, ego dissolution, and relaxation on a scale from 0-100, as has been done in previous studies [5].

Approximately five hours after drug administration, we began preparing participants for EEG measurements. ST was conducted six hours after drug administration. PAS was conducted approximately seven hours after drug administration. A second blood draw for analyzing BDNF was taken eight hours after drug administration. Participants were picked up by a friend or family member once experiments had concluded and drug effects had adequately subsided. They were instructed to complete the 5D-ASC and MEQ before the next appointment, but only after drug effects had completely subsided.

**Follow-up visits.** Participants attended follow-up visits the next day and 6-8 days after drug administration (mean: 7.01). Both visits took place in the afternoon and consisted of ST with questionnaires during the breaks as necessary, followed by the SPSI, PAS, and finally a blood draw for assessing BDNF. Additionally, the motor learning task was completed before all other tests at the visit one day after dosing.

## Software

Computer-based experiments (motor learning, ST) were run using E-Prime (Version 3.03.219, Psychology Software Tools Inc., USA).

## Analysis of evoked theta power

We attempted to replicate a previous exploratory analysis from a study using ST after psilocybin therapy which demonstrated a trend-level increase in evoked theta power two weeks after psilocybin treatment [6]. To this end, we performed a Morlet wavelet transform over the same data used to analyze N1 and P2 ERPs. For each epoch, EEG signals over frontocentral electrodes were decomposed into steps of 0.5Hz for frequencies between 4 and 30 Hz. Raw power estimates (event-related spectral perturbations) were converted into decibels using log_10_ transformation. For each subject and timepoint, we then calculated average power in the theta range (4-8Hz) between 50 and 250 ms after each auditory stimuli to yield a measure of evoked theta power in the relevant time range for N1 and P2 ERPs.

## Questionnaires

Side effects were assessed using a preliminary version of the Swiss Psychedelic Side Effects Inventory (SPSI) [7]. The SPSI records information on 32 side effects relevant to psychedelics and includes information on severity, positive or negative impact, timing, and duration. It also includes a standardized rating of the possibility that a side effect is treatment-related (causality) which is applicable to both placebo and drug conditions even if blinding has been broken. One investigator (AEC or VJD) who had been present on the dosing day conducted the SPSI in interview format both one day and seven days after dosing. One day after dosing, participants reported on side effects they experienced both acutely (within 12 hours of dosing) and post-acutely up until the time of the interview. One week after dosing, participants reported on post-acute side effects occurring since the previous appointment. If any potentially drug-related negative side effects persisted at the final study appointment, investigators followed up with participants until they resolved.

The Cognitive Flexibility Inventory (CFI) and Perceived Stress Scale (PSS) were included to assess subjective post-acute effects. The CFI consists of two factors: *Alternatives,* or the ability to perceive multiple possible explanations for an event and generate multiple possible solutions to a problem, and *Control,* or one’s perceived ability to control difficult situations. The Perceived Stress Scale (PSS) is used to assess subjective psychological stress and participants always answered in relation to the past week.

# Supplemental Results

## Information on missing data

A total of 45 participants were enrolled in the study (Figure S4). One participant was excluded from analysis because of vomiting shortly after LSD ingestion, after which no LSD effects occurred. A second participant was excluded due to dropping out and retrospectively clarifying that inclusion criteria for the study were not met (see also “side effects” below). Additionally, one participant was excluded from TMS analyses because no clear MEPs could be recorded. One participant was excluded from BDNF analyses due to lack of viable blood samples. Additionally, several other participants had missing BDNF data due to difficulties performing blood draws, affecting 1.8% of analyzed samples. Finally, one participant was excluded from motor learning analyses because of a logistic issue with laboratory access at one of the relevant study appointments.

## Side effects

Side effects were analyzed for all 45 participants. LSD increased the number of possibly drug-related side effects reported acutely (β = 6.58, SE = 0.49, *p* < 0.001) and post-acutely within 36 hours after administration (β = 1.40, SE = 0.49, *p* < 0.01), with a trend-level increase during the week after administration (β = 0.88, SE = 0.49, *p* = 0.07) (Table S4, S5). There were no differences in subjective impact scores at any timepoint, and most side effects were rated as having a neutral impact (Figure S5). We followed up with five participants who reported mild but ongoing negative side effects possibly related to LSD at the final study appointment (anxiety (n = 2), reduced stress resilience (n = 1), concentration problems (n = 1), confusion about the meaning of the experience (n = 1). All resolved within 1-3 weeks without professional intervention and none were reported to have lasting negative effects on participants’ well-being. We are not aware of any post-trial adverse events.

Participants could report other side effects than the 32 specified on the SPSI. Other reported acute side effects after LSD were mild and rated as having a neutral impact, including brief shortness of breath (n = 2, presumably related to anxiety); back pain, difficulty moving, nosebleed, metal taste in the mouth, buzzing sounds in the ears, increased need to urinate, increased hair shedding (all n = 1). Other reported post-acute side effects were also mild and included altered perception of other people (n = 3); changes in the menstrual cycle (n = 2); reduced stress resilience, reduced insecurity, nosebleed (same participant as above), pale complexion, increased thoughts about what is important in life (all n = 1). All but one (reduced stress resilience) were described as having neutral impact.

Finally, one participant dropped out and was excluded from analysis after a serious adverse event (SAE) which was deemed to have a possible relationship to LSD. The participant in question received LSD on his first dosing day and reported mostly positive effects. Two investigators were present (AEC and a psychiatrist) who noted no unusual or concerning LSD effects. At follow-ups, the participant reported no notable adverse effects apart from mild fatigue. However, two weeks after receiving LSD, he informed the study team that he could not complete his remaining three study appointments due to voluntary in-patient hospitalization for a depressive episode. He clarified that he had been experiencing depressive symptoms since before entering the study and that some of the statements about his current mental health given during screening had not been entirely accurate, for which he expressed remorse. He further explained the hospitalization by stating that the LSD session had motivated him to face his condition and seek treatment, and he emphasized that the LSD session had neither improved nor worsened his symptoms. The participant was entirely cooperative with all required reporting procedures and was discharged after two weeks of inpatient care. The study team followed up again after an additional month, at which time he reported that he was receiving outpatient treatment and showing overall improvement.

# Supplemental Tables and Figures

*Table S1. Participant demographics (N = 43). Data on previous psychedelic experiences reflects answers regarding serotonergic psychedelics, excluding microdosing.*

| **Characteristic** | **Mean (SD)** |
| --- | --- |
| Age (years) | 30.4 (8.4) |
| Body mass index | 22.1 (2.3) |
| Education (years) | 18.2 (2.6) |
|  | **N (%)** |
| Sex | |
| Female | 24 (55.8%) |
| Male | 19 (44.2%) |
| Any previous psychedelic experiences | 15 (35%) |
| If yes: how many? | 2.4 (range 1-6) |

*Table S2. Hourly ratings of subjective drug effects after drug intake, as well as questionnaire scores for the 5-Dimensional Altered State of Consciousness Questionnaire (5D-ASC) and Mystical Experience Questionnaire (MEQ). Statistics indicate contrasts between placebo (reference level) and LSD. N = 43.*

| **Rating** | **Hour** | **Placebo**  **mean (SD)** | **LSD**  **mean (SD)** | **Estimate** | **SE** | **z** | ***p*** |
| --- | --- | --- | --- | --- | --- | --- | --- |
| **Intensity** | 0 | 0.00 (0.00) | 0.00 (0.00) | 0 | 2.06 | 0 | 1 |
|  | 1 | 6.33 (7.46) | 65.88 (27.62) | 62.45 | 2.06 | 30.3 | <.001 |
|  | 2 | 9.14 (9.55) | 88.70 (18.67) | 83.61 | 2.06 | 40.56 | <.001 |
|  | 3 | 8.09 (11.21) | 86.56 (16.99) | 81.84 | 2.06 | 39.7 | <.001 |
|  | 4 | 4.95 (7.21) | 68.84 (22.03) | 66.31 | 2.06 | 32.17 | <.001 |
|  | 5 | 3.35 (5.37) | 55.81 (24.49) | 52.12 | 2.06 | 25.28 | <.001 |
|  | 6 | 1.30 (2.65) | 34.52 (20.74) | 30.77 | 2.07 | 14.83 | <.001 |
|  | 7 | 0.44 (1.72) | 24.38 (20.03) | 19.94 | 2.07 | 9.61 | <.001 |
|  | 8 | 0.51 (2.14) | 17.43 (17.68) | 13.41 | 2.07 | 6.47 | <.001 |
| **Good drug effect** | 0 | 0.00 (0.00) | 0.00 (0.00) | 0 | 2.32 | 0 | 1 |
|  | 1 | 6.56 (10.29) | 65.98 (27.38) | 64.62 | 2.32 | 27.82 | <.001 |
|  | 2 | 9.35 (13.71) | 81.58 (23.56) | 78.88 | 2.32 | 33.96 | <.001 |
|  | 3 | 8.49 (13.76) | 77.14 (20.94) | 73.43 | 2.34 | 31.42 | <.001 |
|  | 4 | 4.42 (6.53) | 64.44 (26.62) | 63.04 | 2.32 | 27.14 | <.001 |
|  | 5 | 2.67 (4.77) | 53.57 (27.78) | 52.93 | 2.34 | 22.64 | <.001 |
|  | 6 | 1.12 (2.46) | 37.98 (27.47) | 33.35 | 2.34 | 14.27 | <.001 |
|  | 7 | 0.42 (1.67) | 28.57 (26.65) | 21.01 | 2.34 | 8.99 | <.001 |
|  | 8 | 0.40 (1.71) | 22.98 (28.20) | 14.52 | 2.34 | 6.21 | <.001 |
| **Bad drug effect** | 0 | 0.00 (0.00) | 0.00 (0.00) | 0 | 0.36 | 0 | 1 |
|  | 1 | 0.44 (1.20) | 8.95 (14.08) | 2.92 | 0.36 | 8.16 | <.001 |
|  | 2 | 0.19 (0.63) | 13.28 (24.40) | 2.24 | 0.36 | 6.27 | <.001 |
|  | 3 | 1.67 (9.90) | 9.33 (16.60) | 1.73 | 0.36 | 4.8 | <.001 |
|  | 4 | 0.56 (2.13) | 8.98 (17.23) | 1.41 | 0.36 | 3.93 | <.001 |
|  | 5 | 0.14 (0.52) | 7.95 (19.09) | 1.32 | 0.36 | 3.68 | <.001 |
|  | 6 | 0.16 (0.65) | 5.17 (10.02) | 1.64 | 0.36 | 4.55 | <.001 |
|  | 7 | 0.05 (0.21) | 4.71 (8.65) | 1.52 | 0.36 | 4.22 | <.001 |
|  | 8 | 0.05 (0.30) | 5.43 (11.24) | 1.49 | 0.36 | 4.13 | <.001 |
| **Ego**  **dissolution** | 0 | 0.00 (0.00) | 0.00 (0.00) | 0 | 1.95 | 0 | 1 |
|  | 1 | 1.33 (3.94) | 29.07 (31.27) | 17.5 | 1.95 | 8.99 | <.001 |
|  | 2 | 1.05 (2.74) | 56.07 (39.37) | 67.88 | 1.95 | 34.86 | <.001 |
|  | 3 | 0.23 (1.07) | 54.67 (34.27) | 57.14 | 1.96 | 29.17 | <.001 |
|  | 4 | 0.12 (0.76) | 39.77 (32.95) | 33.26 | 1.95 | 17.08 | <.001 |
|  | 5 | 0.00 (0.00) | 27.57 (26.40) | 21.05 | 1.96 | 10.75 | <.001 |
|  | 6 | 0.00 (0.00) | 13.17 (17.84) | 9.72 | 1.96 | 4.96 | <.001 |
|  | 7 | 0.12 (0.76) | 8.50 (13.92) | 5.97 | 1.96 | 3.05 | 0.0023 |
|  | 8 | 0.12 (0.76) | 4.55 (7.96) | 4.34 | 1.96 | 2.22 | 0.0266 |
| **Relaxation** | 0 | 71.86 (21.99) | 66.40 (19.64) | 6.36 | 2.95 | 2.16 | 0.031 |
|  | 1 | 83.95 (15.68) | 72.40 (26.07) | 10.26 | 2.95 | 3.48 | <.001 |
|  | 2 | 89.63 (10.63) | 82.44 (23.03) | 5.16 | 2.95 | 1.75 | 0.0801 |
|  | 3 | 89.14 (11.50) | 80.71 (23.70) | 6.23 | 2.97 | 2.1 | 0.0358 |
|  | 4 | 85.23 (13.76) | 80.21 (23.12) | 1.29 | 2.95 | 0.44 | 0.6604 |
|  | 5 | 83.49 (13.69) | 78.00 (21.26) | 1.77 | 2.97 | 0.6 | 0.5498 |
|  | 6 | 82.77 (14.04) | 80.00 (17.91) | 1.69 | 2.97 | 0.57 | 0.5689 |
|  | 7 | 80.37 (16.08) | 80.71 (20.11) | 1.95 | 2.97 | 0.66 | 0.5109 |
|  | 8 | 78.93 (15.15) | 81.12 (20.35) | 3.77 | 2.97 | 1.27 | 0.2034 |
| **5D-ASC** | | | | | | | |
| Oceanic Boundlessness | | 4.48 (6.33) | 55.80 (25.43) | 52.78 | 3.8 | 13.89 | <.001 |
| Anxious Ego Dissolution | | 1.13 (2.16) | 28.96 (23.17) | 25.92 | 3.8 | 6.82 | <.001 |
| Visionary Restructuralization | | 4.21 (7.29) | 56.43 (25.16) | 53.21 | 3.8 | 14 | <.001 |
| Auditory Alterations | | 1.81 (4.32) | 24.45 (21.80) | 21.01 | 3.8 | 5.53 | <.001 |
| Vigilance Reduction | | 18.75 (17.17) | 48.47 (25.03) | 30.61 | 3.8 | 8.06 | <.001 |
| **MEQ-30** | | | | | | | |
| Mystical | | 4.00 (6.63) | 51.94 (25.78) | 50.25 | 3.03 | 16.61 | <.001 |
| Positive Mood | | 15.43 (14.11) | 67.05 (21.80) | 53.4 | 3.03 | 17.64 | <.001 |
| Transcendence | | 5.97 (8.25) | 66.67 (24.83) | 63.72 | 3.03 | 21.05 | <.001 |
| Ineffability | | 5.12 (8.71) | 84.19 (19.68) | 81.09 | 3.03 | 26.8 | <.001 |

*Table S3. Percentage of participants who gave each possible rating of lasting LSD effects on subjective well-being at the final study visit, divided by order (LSD or placebo first). Participants most commonly reported mildly or moderately positive lasting effects on well-being, and no one reported negative lasting effects. N = 43.*

| **“Do you have the feeling that the LSD experience led to**  **changes in your personal well-being or life satisfaction?”** | | |
| --- | --- | --- |
|  | **LSD first**  **(n = 21)** | **Placebo first**  **(n = 22)** |
| Strong increase | 14.29% | 22.73% |
| Moderate increase | 23.81% | 22.73% |
| Slight increase | 38.1% | 36.36% |
| No change | 23.81% | 18.18% |
| Slight decrease | 0% | 0% |
| Moderate decrease | 0% | 0% |
| Strong decrease | 0% | 0% |
| *Time since LSD experience (days)* | *72.57 (62.41)* | *6.95 (0.56)* |

*Table S4. Impact of LSD on side effects recorded with the Swiss Psychedelic Side Effects Inventory (SPSI). Data show the number of possibly treatment-related side effects and ratings of their subjective impact at three timepoints after drug administration. Impact ratings range from -2 (very negative) to +2 (very positive). Statistics indicate results of linear mixed effects models with placebo as the reference level. N = 45.*

| **Timepoint** | **Placebo**  **mean (SD)** | **LSD**  **mean (SD)** | **Estimate** | **SE** | **z** | ***p*** |
| --- | --- | --- | --- | --- | --- | --- |
| **Number of side effects** | | | | | | |
| Acute (<12h) | 2.64 (2.20) | 9.38 (4.22) | 6.58 | 0.49 | 13.36 | <.001 |
| Post-acute (12-36h) | 3.96 (3.01) | 5.76 (3.80) | 1.4 | 0.49 | 2.85 | 0.004 |
| Prolonged (36h - 1 week) | 1.40 (1.89) | 2.29 (2.20) | 0.88 | 0.49 | 1.78 | 0.075 |
| **Subjective impact of side effects** | | | | | | |
| Acute (<12h) | 0.00 (0.37) | 0.33 (1.43) | 0 | 0 | 1.2 | 0.231 |
| Post-acute (12-36h) | -0.56 (1.42) | -0.31 (1.08) | 0 | 0 | 1.07 | 0.286 |
| Prolonged (36h - 1 week) | -0.27 (1.12) | -0.31 (0.79) | 0 | 0 | -1.13 | 0.258 |

*Table S5. Percentage of participants reporting each side effect from the Swiss Psychedelic Side Effects Inventory (SPSI) arranged in the order of most frequent acute effects after LSD. Only side effects rated as at least possibly drug-related are shown. See also Figure S2 for visualization of impact ratings from the SPSI. N = 45.*

| **Side effect** | **LSD acute (<12h)** | **LSD post-acute**  **(12-36h)** | **LSD prolonged**  **(36h-7d)** | **Placebo acute (<12h)** | **Placebo post-acute**  **(12-36h)** | **Placebo prolonged**  **(36h-7d)** |
| --- | --- | --- | --- | --- | --- | --- |
| Vision changes | 95.56 | 15.56 | 11.11 | 33.33 | 4.44 | 0 |
| Difficulty concentrating | 71.11 | 35.56 | 17.78 | 20 | 26.67 | 4.44 |
| Appetite changes | 66.67 | 28.89 | 15.56 | 6.67 | 8.89 | 2.22 |
| Muscle shaking, tightness | 64.44 | 11.11 | 8.89 | 6.67 | 20 | 8.89 |
| Hot, cold, sweaty | 60 | 15.56 | 2.22 | 17.78 | 4.44 | 0 |
| Nausea, vomiting | 60 | 11.11 | 0 | 4.44 | 15.56 | 2.22 |
| Anxious, fearful, panicked | 44.44 | 40 | 17.78 | 11.11 | 13.33 | 6.67 |
| Fatigue, tiredness | 44.44 | 77.78 | 60 | 57.78 | 64.44 | 42.22 |
| Headache | 42.22 | 60 | 17.78 | 28.89 | 55.56 | 11.11 |
| Confusion, disorientation | 40 | 11.11 | 4.44 | 0 | 4.44 | 2.22 |
| Depersonalization | 40 | 0 | 0 | 0 | 2.22 | 0 |
| Derealization | 37.78 | 2.22 | 0 | 0 | 0 | 0 |
| Bloating, diarrhea | 33.33 | 26.67 | 0 | 6.67 | 28.89 | 4.44 |
| Dizziness, faintness | 33.33 | 8.89 | 0 | 17.78 | 8.89 | 2.22 |
| Memory problems | 33.33 | 6.67 | 6.67 | 11.11 | 6.67 | 2.22 |
| Heart racing, chest pressure | 31.11 | 13.33 | 0 | 15.56 | 11.11 | 2.22 |
| Restless, irritable | 28.89 | 24.44 | 8.89 | 6.67 | 24.44 | 15.56 |
| Hearing changes | 22.22 | 8.89 | 2.22 | 6.67 | 2.22 | 0 |
| Fear of going insane | 20 | 8.89 | 0 | 0 | 0 | 0 |
| Lonely, isolated | 17.78 | 13.33 | 11.11 | 4.44 | 4.44 | 2.22 |
| Negative thoughts about self | 15.56 | 0 | 2.22 | 0 | 4.44 | 0 |
| Sad, mournful, depressed | 11.11 | 17.78 | 8.89 | 6.67 | 8.89 | 2.22 |
| Apathetic, empty | 8.89 | 6.67 | 2.22 | 2.22 | 13.33 | 2.22 |
| Distressing memories | 4.44 | 6.67 | 4.44 | 0 | 0 | 0 |
| Paranoia | 4.44 | 0 | 0 | 0 | 0 | 0 |
| Sleep difficulties | 4.44 | 91.11 | 17.78 | 0 | 51.11 | 24.44 |
| Existential distress | 2.22 | 0 | 0 | 0 | 0 | 0 |
| Desire to take again | 0 | 0 | 0 | 0 | 0 | 0 |
| Elated, excitable, impulsive | 0 | 0 | 0 | 0 | 0 | 0 |
| Psychedelic flashbacks | 0 | 33.33 | 8.89 | 0 | 11.11 | 2.22 |
| Suicidality | 0 | 0 | 0 | 0 | 0 | 0 |
| Thought disorder | 0 | 0 | 0 | 0 | 0 | 0 |

*Table S6. Main effects from mixed effects models demonstrating that there was no effect of sensory tetanization (ST; in bold) on amplitude of N1 (top) or P2 (bottom) event-related potentials (ERPs). Post-hoc contrasts confirmed that ST did not potentiate ERPs at at any visit. T = time of drug intake. N = 43.*

| **Fixed effect** | **Coefficient** | **SE** | **CI low** | **CI high** | **t** | **df** | ***p*** |
| --- | --- | --- | --- | --- | --- | --- | --- |
| **N1 model** | | | | | | | |
| drug | -0.57 | 0.15 | -0.87 | -0.27 | -3.72 | 987 | <0.001 |
| visit T+1 | -0.30 | 0.16 | -0.60 | 0.01 | -1.92 | 987 | 0.055 |
| visit T+7 | -0.40 | 0.15 | -0.71 | -0.10 | -2.62 | 987 | 0.009 |
| **ST** | **0.05** | **0.13** | **-0.20** | **0.30** | **0.39** | **987** | **0.700** |
| baseline amplitude | 0.92 | 0.06 | 0.80 | 1.04 | 14.51 | 987 | <0.001 |
| order | 0.10 | 0.19 | -0.29 | 0.48 | 0.49 | 987 | 0.625 |
| exercise | 0.03 | 0.03 | -0.03 | 0.10 | 1.03 | 987 | 0.302 |
| sleep | 0.03 | 0.02 | -0.01 | 0.07 | 1.42 | 987 | 0.156 |
| **P2 model** | | | | | | | |
| drug | 1.55 | 0.22 | 1.12 | 1.98 | 7.05 | 987 | <0.001 |
| visit T+1 | 0.97 | 0.22 | 0.53 | 1.41 | 4.34 | 987 | <0.001 |
| visit T+7 | 1.75 | 0.22 | 1.31 | 2.18 | 7.86 | 987 | <0.001 |
| **ST** | **0.15** | **0.18** | **-0.21** | **0.51** | **0.82** | **987** | **0.414** |
| baseline amplitude | 0.63 | 0.07 | 0.49 | 0.78 | 8.85 | 987 | <0.001 |
| order | 0.03 | 0.31 | -0.58 | 0.63 | 0.09 | 987 | 0.929 |
| exercise | -0.03 | 0.05 | -0.12 | 0.06 | -0.62 | 987 | 0.533 |
| sleep | -0.01 | 0.03 | -0.07 | 0.05 | -0.32 | 987 | 0.746 |

*Table S7. Effects of LSD on amplitude of N1 and P2 auditory event-related potentials (ERPs) after auditory stimuli during each recording session. Statistics show contrasts between placebo (reference) and LSD at each timepoint. T = time of drug intake. N = 43.*

| **ERP** | **Timepoint** | **Placebo mean (SD)** | **LSD mean (SD)** | **Estimate** | **SE** | **z** | ***p*** |
| --- | --- | --- | --- | --- | --- | --- | --- |
| N1 | T + 6h | -3.90 (1.66) | -3.42 (1.70) | 0.5 | 0.08 | 6.45 | <.001 |
| N1 | T + 1d | -3.66 (1.71) | -3.76 (1.64) | -0.01 | 0.08 | -0.17 | 0.869 |
| N1 | T + 7d | -3.72 (1.69) | -3.79 (1.72) | -0.04 | 0.08 | -0.56 | 0.579 |
| P2 | T + 6h | 4.65 (2.28) | 3.07 (1.69) | -1.33 | 0.11 | -12.07 | <.001 |
| P2 | T + 1d | 4.52 (1.83) | 4.23 (1.99) | -0.32 | 0.11 | -2.92 | 0.004 |
| P2 | T + 7d | 4.46 (1.80) | 4.63 (2.07) | -0.02 | 0.11 | -0.22 | 0.825 |

*Table S8. Results of contrasts from linear mixed effects models assessing the effects of LSD on evoked theta power at each visit. We found no effect of LSD on evoked theta power. T = time of drug intake. N = 43.*

| **Timepoint** | **Placebo**  **mean (SD)** | **LSD**  **mean (SD)** | **Estimate** | **SE** | **z** | ***p*** |
| --- | --- | --- | --- | --- | --- | --- |
| T + 6h | 2.85 (2.48) | 3.14 (2.67) | 0.33 | 0.23 | 1.44 | 0.151 |
| T + 1d | 2.98 (2.37) | 2.94 (2.27) | -0.11 | 0.23 | -0.46 | 0.647 |
| T + 7d | 2.90 (2.49) | 3.08 (2.69) | 0.07 | 0.23 | 0.32 | 0.751 |

*Table S9. Results from mixed effects models demonstrating that there was no overall effect of paired associative stimulation (PAS, in bold) on amplitude of motor-evoked potentials (MEPs). Post-hoc contrasts confirmed that there was no potentiation effect on MEPs at any visit. T = time of drug intake. N = 42.*

| **Fixed effect** | **Coefficient** | **SE** | **CI low** | **CI high** | **t** | **df** | ***p*** |
| --- | --- | --- | --- | --- | --- | --- | --- |
| drug | -76.21 | 52.21 | -178.68 | 26.27 | -1.46 | 923 | 0.145 |
| visit T+1 | -107.19 | 53.10 | -211.40 | -2.99 | -2.02 | 923 | 0.044 |
| visit T+7 | -54.91 | 53.04 | -159.00 | 49.18 | -1.04 | 923 | 0.301 |
| **PAS** | **7.44** | **43.87** | **-78.65** | **93.53** | **0.17** | **923** | **0.865** |
| order | -67.04 | 62.33 | -189.37 | 55.29 | -1.08 | 923 | 0.282 |
| exercise | 6.10 | 11.86 | -17.18 | 29.38 | 0.51 | 923 | 0.607 |
| sleep | 7.71 | 6.93 | -5.89 | 21.32 | 1.11 | 923 | 0.266 |
| TMS count | 1.23 | 0.30 | 0.63 | 1.82 | 4.04 | 923 | <0.001 |
| baseline amplitude | 0.76 | 0.09 | 0.59 | 0.93 | 8.79 | 923 | <0.001 |
| **drug x PAS** | **16.12** | **60.08** | **-101.79** | **134.02** | **0.27** | **923** | **0.789** |

*Table S10. Effects of LSD on amplitudes and latencies of motor-evoked potentials (MEPs). MEP amplitude was acutely increased under LSD and decreased one day after LSD. Latency was acutely decreased under LSD. Statistics show contrasts between placebo (reference) and LSD at each timepoint. T = time of drug intake. N = 42.*

| **MEP**  **Parameter** | **Timepoint** | **Placebo**  **mean (SD)** | **LSD**  **mean (SD)** | **Estimate** | **SE** | **z** | ***p*** |
| --- | --- | --- | --- | --- | --- | --- | --- |
| amplitude | T + 7h | 775.16 (403.29) | 808.60 (375.73) | 64.4 | 26.63 | 2.42 | 0.016 |
| amplitude | T + 1d | 874.35 (397.24) | 775.24 (375.80) | -80.76 | 26.11 | -3.09 | 0.002 |
| amplitude | T + 7d | 829.02 (394.67) | 787.45 (370.08) | -40.71 | 25.4 | -1.6 | 0.109 |
| latency | T + 7h | 23781.99 (2190.11) | 22913.53 (2149.46) | -775.19 | 119.75 | -6.47 | <.001 |
| latency | T + 1d | 23372.61 (1941.89) | 23372.69 (2004.43) | -104.7 | 117.58 | -0.89 | 0.373 |
| latency | T + 7d | 23238.02 (2169.24) | 23281.63 (2142.94) | 42.69 | 114.4 | 0.37 | 0.709 |

*Table S11. Effects of LSD on plasma and serum brain-derived neurotrophic factor (BDNF) at four timepoints before and after dosing. Statistics show contrasts between placebo (reference) and LSD at each timepoint. T = time of drug intake. N = 42.*

| **Timepoint** | **Blood**  **fraction** | **Placebo**  **mean (SD)** | **LSD**  **mean (SD)** | **Estimate** | **SE** | **z** | ***p*** |
| --- | --- | --- | --- | --- | --- | --- | --- |
| T - 0.5h | serum | 22.97 (5.68) | 23.55 (7.48) | 0.49 | 1.21 | 0.41 | 0.685 |
| T + 8h | serum | 22.73 (5.85) | 23.92 (7.57) | 1.06 | 1.23 | 0.86 | 0.389 |
| T + 1d | serum | 23.93 (8.03) | 23.93 (7.65) | -0.63 | 1.22 | -0.52 | 0.605 |
| T + 7d | serum | 25.97 (7.43) | 26.36 (8.81) | -0.37 | 1.19 | -0.31 | 0.757 |
| T - 0.5h | plasma | 1.93 (1.87) | 1.97 (1.35) | 0.26 | 0.16 | 1.61 | 0.107 |
| T + 8h | plasma | 1.55 (1.07) | 1.77 (1.75) | 0.07 | 0.17 | 0.39 | 0.698 |
| T + 1d | plasma | 2.06 (1.92) | 1.75 (1.34) | -0.19 | 0.17 | -1.11 | 0.267 |
| T + 7d | plasma | 1.75 (1.41) | 1.98 (1.69) | 0.03 | 0.16 | 0.2 | 0.844 |

*Table S12. Effects of LSD on typing speed improvement during a motor learning task one day after drug administration. P2-P10 indicate practice blocks assessing online learning; T1 and T2 indicate blocks after 10 and 80 minutes of rest assessing offline learning. Data show improvement in each block compared to the previous practice block. More negative values indicate greater improvement. Note that the first practice block (P1) is not shown because it has no difference to a previous block. Statistics show results of linear mixed effects models with contrasts between placebo (reference level) and LSD. N = 42.*

| **Block** | **Placebo**  **mean (SD)** | **LSD**  **mean (SD)** | **Estimate** | **SE** | **z** | ***p*** |
| --- | --- | --- | --- | --- | --- | --- |
| P2 | -123.83 (144.59) | -119.35 (177.95) | 11.85 | 12.56 | 0.94 | 0.346 |
| P3 | -98.30 (93.60) | -82.05 (92.43) | 20.03 | 12.56 | 1.59 | 0.111 |
| P4 | -66.44 (63.99) | -52.76 (74.30) | 15.42 | 12.56 | 1.23 | 0.220 |
| P5 | -50.79 (55.10) | -54.36 (64.97) | -2.57 | 12.56 | -0.2 | 0.838 |
| P6 | -40.89 (44.59) | -48.15 (46.73) | -8.03 | 12.56 | -0.64 | 0.523 |
| P7 | -38.66 (41.18) | -35.18 (35.35) | 2.52 | 12.56 | 0.2 | 0.841 |
| P8 | -34.65 (29.75) | -32.50 (36.01) | 1.76 | 12.56 | 0.14 | 0.889 |
| P9 | -26.91 (26.07) | -25.15 (26.76) | 0.37 | 12.56 | 0.03 | 0.977 |
| P10 | -24.46 (24.57) | -27.87 (27.07) | -3.57 | 12.56 | -0.28 | 0.776 |
| T1 | -283.54 (222.94) | -332.33 (341.63) | -5.71 | 12.56 | -0.45 | 0.650 |
| T2 | -254.40 (227.08) | -347.86 (324.61) | -36.98 | 12.56 | -2.94 | 0.003 |

*Table S13. Results of Spearman correlations assessing relationships between behavioral and neurophysiological outcomes, with correction for false discovery rate. No significant correlations were found. Correlations with offline motor learning used improvement in the test block after 80 minutes of rest. BDNF = brain-derived neurotrophic factor, CFI = Cognitive Flexibility Inventory, MEP = motor-evoked potential, PSS = Perceived Stress Scale, T = time of drug intake. N = 43.*

| **Outcome 1** | **Outcome 2** | **Timepoint** | **Spearman's ρ** | ***p*** | ***p* (corrected)** |
| --- | --- | --- | --- | --- | --- |
| Motor learning | MEP amplitude | T+7h | 0.06 | 0.59 | 0.77 |
| Motor learning | MEP latency | T+7h | 0.18 | 0.12 | 0.56 |
| Motor learning | MEP amplitude | T+1d | 0.09 | 0.44 | 0.77 |
| Motor learning | P2 amplitude | T+1d | -0.08 | 0.47 | 0.77 |
| Motor learning | Serum BDNF | T+1d | -0.06 | 0.62 | 0.77 |
| Motor learning | Plasma BDNF | T+1d | -0.02 | 0.83 | 0.92 |
| PSS | Serum BDNF | T+7d | 0.00 | 0.97 | 0.97 |
| PSS | Plasma BDNF | T+7d | 0.10 | 0.39 | 0.77 |
| CFI Alternatives | Serum BDNF | T+7d | -0.15 | 0.17 | 0.56 |
| CFI Alternatives | Plasma BDNF | T+7d | 0.19 | 0.09 | 0.56 |

*Table S14. Scores on two subscales of the Cognitive Flexibility Inventory (CFI) and Perceived Stress Scale (PSS) one week after LSD and placebo. LSD significantly increased scores on the Alternatives subscale of the CFI and significantly decreased scores on the PSS. Statistics show contrasts between placebo (reference) and LSD one week after dosing from mixed effects models including baseline scores as covariates. N = 43.*

| **Questionnaire** | **Placebo**  **mean (SD)** | **LSD**  **mean (SD)** | **Estimate** | **SE** | **z** | ***p*** |
| --- | --- | --- | --- | --- | --- | --- |
| CFI Alternatives | 73.93 (8.46) | 76.00 (6.85) | 1.92 | 0.63 | 3.04 | 0.002 |
| CFI Control | 37.56 (6.80) | 37.07 (6.90) | -0.53 | 0.67 | -0.8 | 0.424 |
| Perceived Stress Scale | 23.23 (5.69) | 21.21 (4.96) | -1.94 | 0.91 | -2.14 | 0.032 |

*
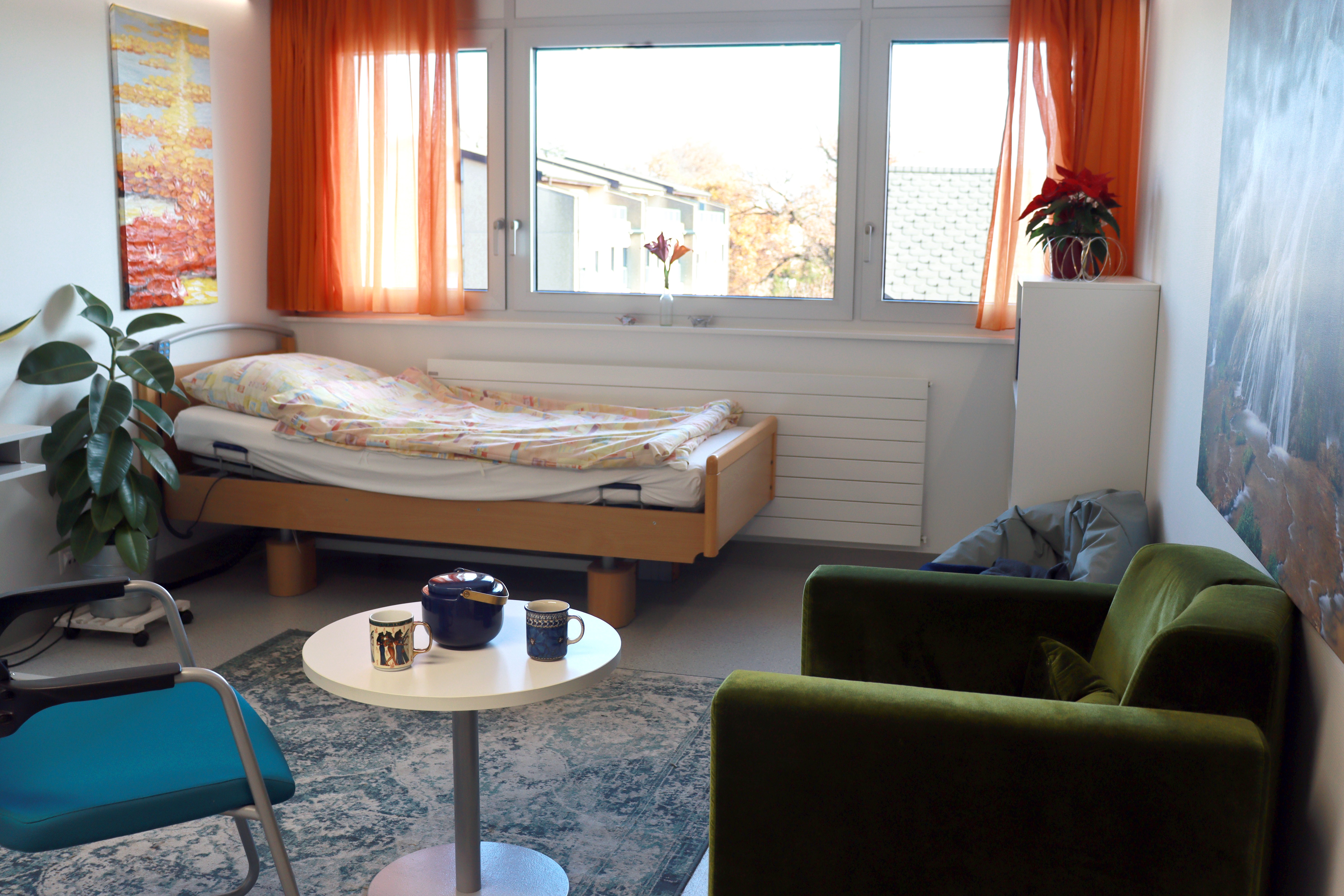
*

*Figure S1. The room used for dosing sessions in the study. Photo credit David Elmiger.*

*
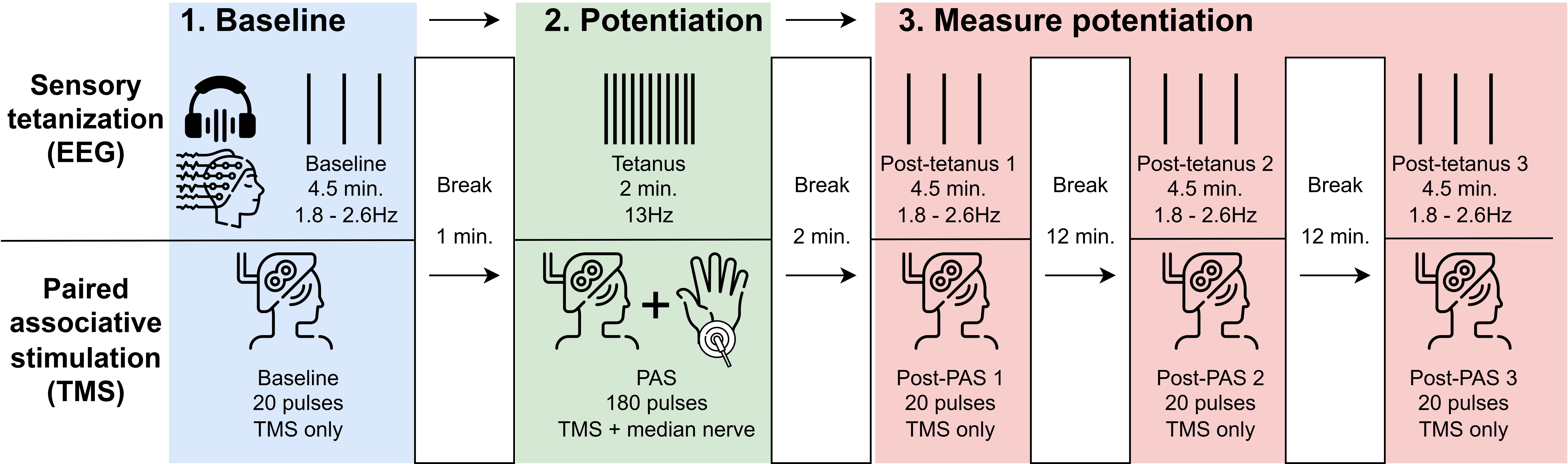
*

*Figure S2. Procedures for assessing LTP-like plasticity using two stimulation-based methods. Sensory tetanization (ST, top) uses auditory tones played at 13Hz to potentiate auditory event-related potentials measured with electroencephalography (EEG). Paired associative stimulation (PAS, bottom) uses a combination of transcranial magnetic stimulation (TMS) and median nerve stimulation to potentiate motor-evoked potentials (MEPs) measured using electromyography. Both procedures first involve measuring baseline neural responses to the stimulus, namely auditory tones or single TMS pulses (1). Next, they aim to potentiate that response using brain stimulation (2). Finally, the effect of potentiation on neural signals is measured for approximately 30 minutes afterward (3). Potentiated signals are thought to reflect LTP-like plasticity.*

*
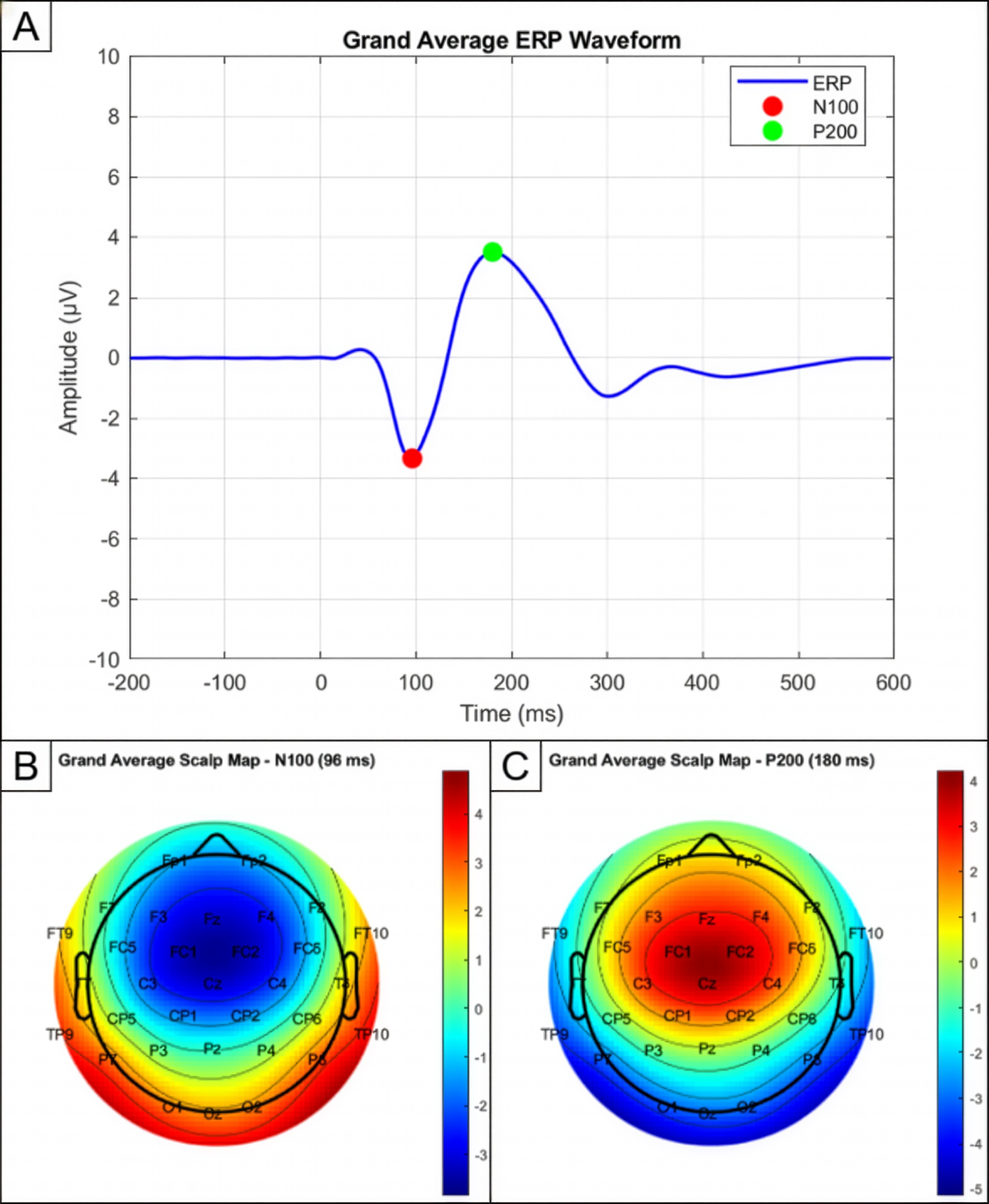
*

*Figure S3. Grand average ERP waveforms (A) and scalp signals for auditory N1 (B) and P2 (C) event-related potentials across all participants and visits.*

Excluded from analysis (emesis of LSD dose) (n= 1)

Analysis

Intervention

Analysed (n= 43)

Completed the study (n= 44)

Randomised (n= 45)

Enrolment

Assessed for eligibility (n= 83)

Excluded (n= 38)

Not meeting inclusion criteria (n= 22)

Scheduling issues (n= 7)

Other reasons (n= 9)

Did not complete the study (n= 1)

Dropped out (retrospective determination of ineligibility) (n= 1)

*Figure S4. CONSORT flow diagram showing movement of participants through the trial. See supplemental results for details regarding excluded participants.*

*

Figure S5. Acute subjective effects of 100µg LSD and placebo. LSD significantly increased hourly ratings of effect intensity (A), good drug effect (B), bad drug effect (C), and ego dissolution (D). LSD significantly but weakly decreased subjective feelings of relaxation (E). LSD also significantly increased scores on all subscales of the 5-Dimensional Altered States of Consciousness Questionnaire (5D-ASC) (F)and Mystical Experience Questionnaire (MEQ) (G). 5D-ASC and MEQ values are shown as the percentage of the maximum possible score. See Table S1 for summary statistics and results of mixed models. Error bars show SEM. N = 43. ***p < .001. ** p < .01, *p < .05.*

*
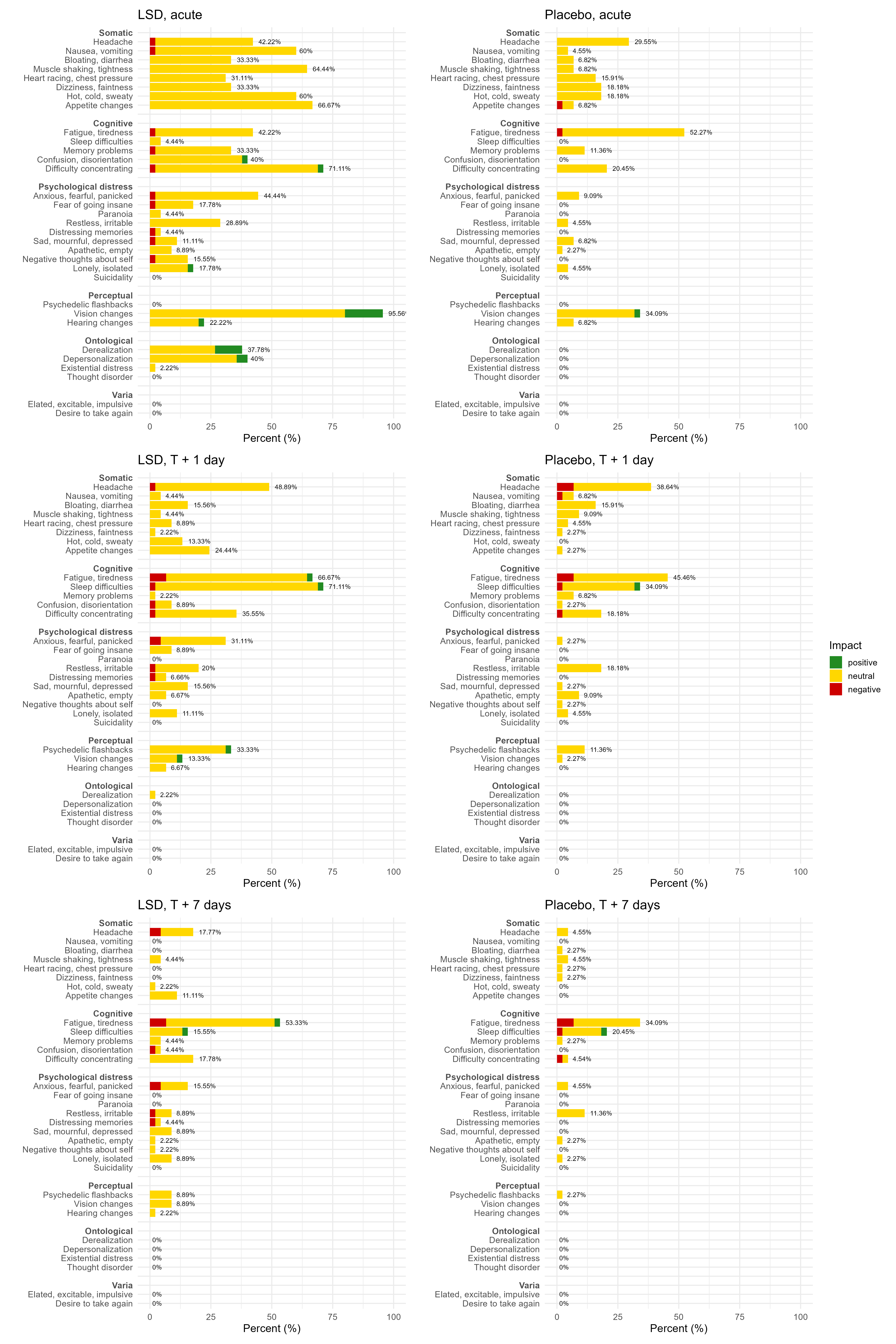
*

*Figure S6. Descriptive graph of subjective impact ratings for possibly drug-related side effects reported on the Swiss Psychedelic Side Effects Inventory (SPSI) for three time periods after administration of LSD and placebo. Impact ratings range from -2 (very negative) to +2 (very positive). Side effects were considered acute if they occurred within 12 hours of drug administration. N = 45.*





*Figure S7. Effects of LSD on auditory event-related potentials (ERPs) before and after sensory tetanization (ST). N = 43. Waveforms (A) show average ERPs before and after ST for each drug and visit. Bar graphs show mean amplitude of N1 and P2 components before and after ST for each drug and visit. P2 amplitude significantly decreased after tetanus in the LSD condition one week after dosing. Error bars show SEM. Only the results of statistical tests related to ST are shown here; see main text for main effects of LSD on auditory ERPs. T = time of drug intake. N = 43. *p < .05.*

*

Figure S8. Auditory ERP amplitudes at all four EEG recording timepoints (one pre-tetanus, three post-tetanus) for each drug and visit. Analysis of linear trends showed that decreases in P2 amplitude one week after dosing were significantly greater after LSD than placebo. This effect was analyzed in post-hoc tests following the significant tetanus effect observed for P2 in the LSD condition at one week; no rationale existed for statistical analysis of other linear trends in ERP amplitudes, which are shown for visual comparison only. See Figure 8 and Table S6 for main effects of LSD on auditory ERP amplitudes. Error bars show SEM. T = time of drug intake. Error bars show SEM. N = 43. *p < .05.*

*
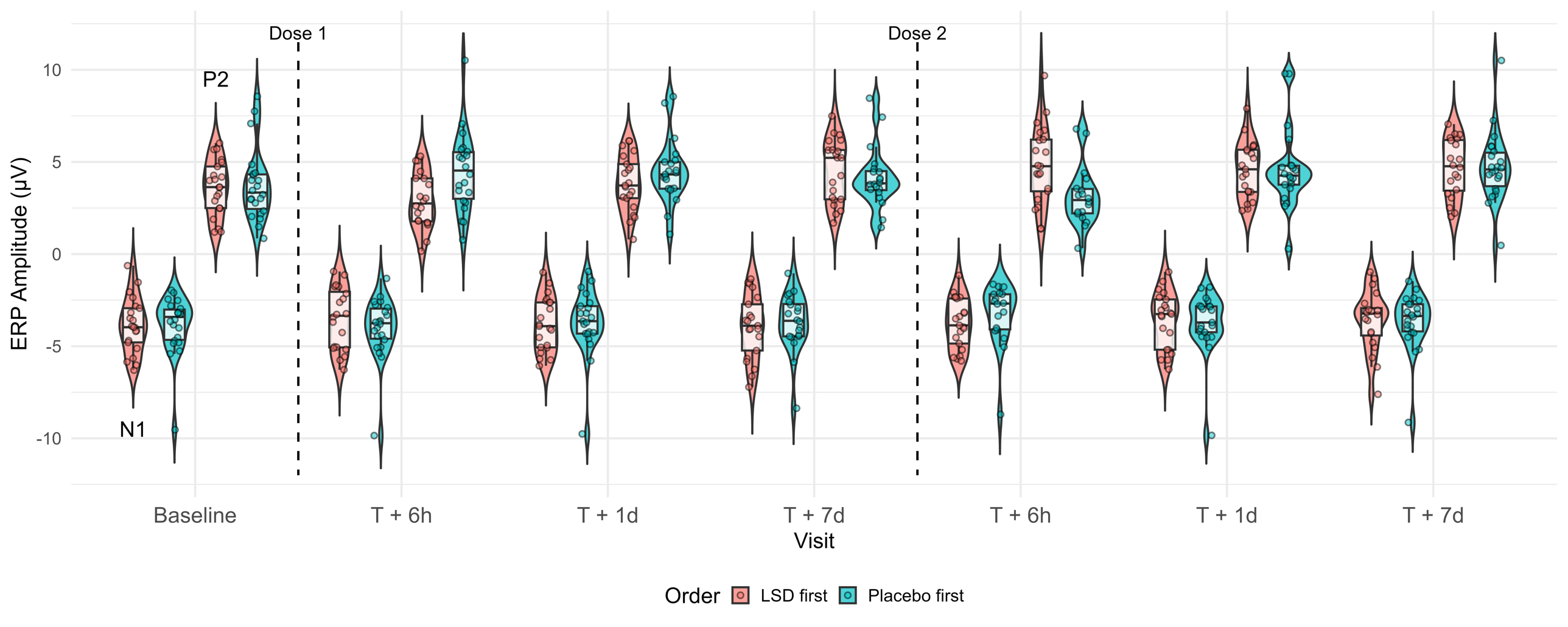
*

*Figure S9. Amplitudes of auditory N1 (lower) and P2 (upper) event-related potentials at each study visit for each treatment order condition. This image shows change over time on repeated measures for descriptive purposes only; see Figure 3 for results of statistical tests pooled by drug condition. Statistical models showed no significant effect of treatment order. ERP = event-related potential, T = time of drug intake. N = 43.*





*Figure S10. Motor-evoked potential amplitudes (upper) and latencies (lower) at each study visit for each treatment order condition. This image shows change over time on repeated measures for descriptive purposes only; see Figure 4 for results of statistical tests pooled by drug condition. Statistical models showed no significant effect of treatment order. MEP = motor-evoked potential, T = time of drug intake. N = 42.*


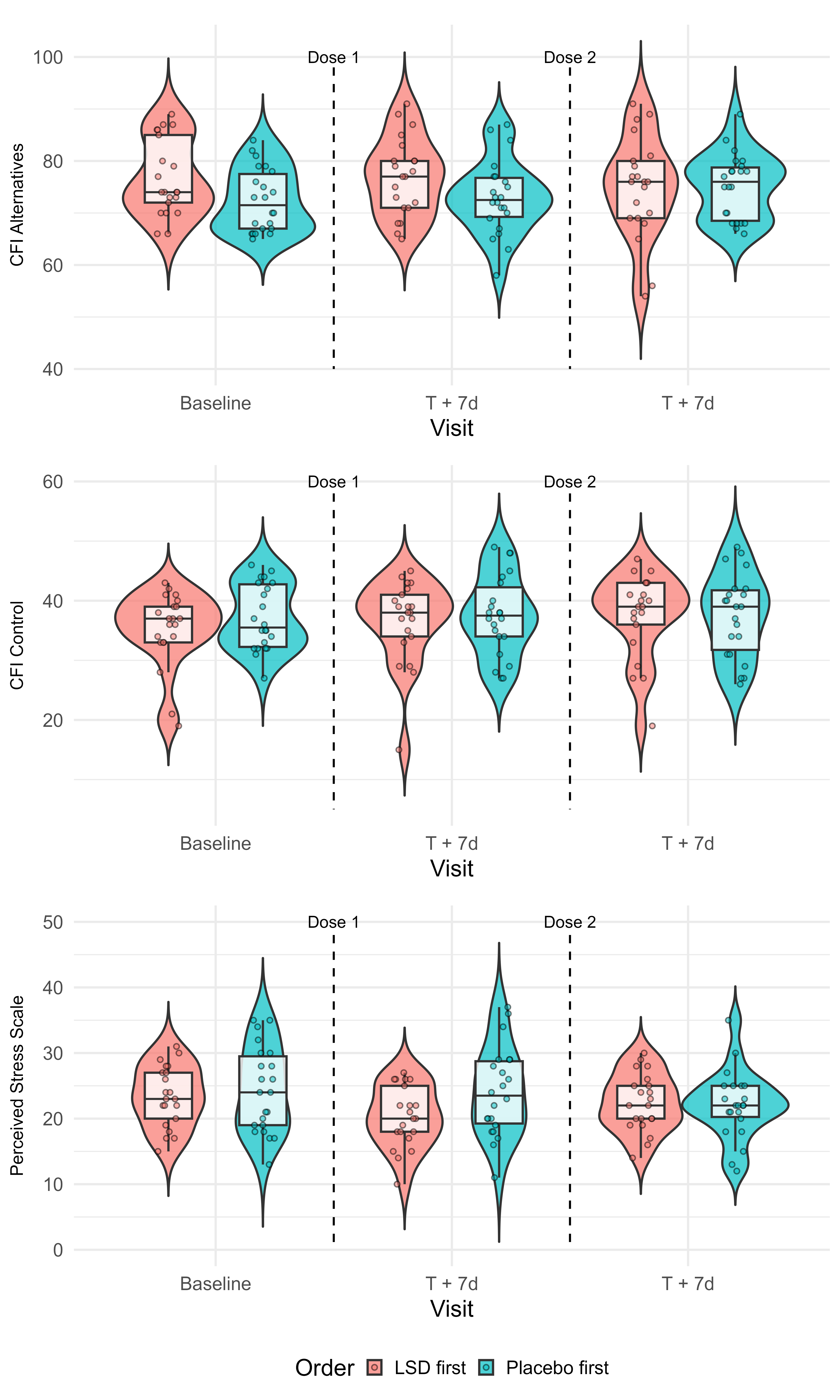


*Figure S11. Scores on the two subscales of the Cognitive Flexibility Inventory, as well as the Perceived Stress Scale, at each study visit for each treatment order condition. This image shows change over time on repeated measures for descriptive purposes only; see Figure 5 for results of statistical tests pooled by drug condition. Statistical models showed no significant effect of treatment order. CFI = Cognitive Flexibility Inventory, T = time of drug intake. N = 43.*

# ReSPCT reporting of preparation and setting for LSD sessions

The following information on the setting surrounding LSD sessions has been prepared with reference to the ReSPCT Guidelines for Reporting of Setting in Psychedelic Clinical Trials [8].

## Physical environment of LSD sessions

Dosing sessions took place indoors in the research wing of a mental health clinic located on the outskirts of Fribourg, Switzerland in a suburban area with views of meadows, trees, apartment buildings, a flower garden, and mountains. Participants had free access to the study room, a balcony, and a single stall bathroom directly across the hall. We aimed to create a comfortable living room-like atmosphere in the study room. In addition to necessary computers and supplies required for tests, the study room contained comfortable furniture (armchair, meditation cushions, bed with adjustable bedframe), wall art featuring natural landscapes, house plants, a rug, other small decorations (e.g. origami, decorative rocks), and a bookshelf with titles related to neuroscience and psychedelics (see Figure 2 of the main text). We avoided objects with overt religious or spiritual connotations and participants were encouraged to bring their own objects if desired.

Several lighting options were available and participants were allowed to adjust them at will (outside of experiments). There was one white fluorescent light on the ceiling with adjustable brightness. There were also two strips of colored lights on the ceiling which could be turned on or off and made to be nearly any color. Participants were given the color remote at the beginning of dosing sessions. Blinds and/or curtains could be closed to darken the room. Participants were given a comfortable blindfold and could bring their own headphones if desired; otherwise music was played over loudspeakers. Participants were encouraged to use blindfolds and listen to music if they desired an intense introspective experience, but this was not required.

Participants could sometimes hear noise from outside or from neighboring clinic rooms, e.g. people talking, cars, or music. The possibility of hearing these noises was discussed beforehand to set expectations, and participants were encouraged to shut windows or wear headphones if noises were bothersome. There were no disturbances which compromised participants’ privacy, though participants were advised that they might see other (friendly) researchers if they stepped into the hallway (e.g. to go to the balcony or bathroom). Participants on LSD were never unaccompanied except when in the bathroom, in which case a facilitator waited outside.

## Study team

At least one member of the facilitator team (AEC) was kept consistent throughout all preparation, dosing and integration sessions, and participants had the same facilitators at all appointments whenever scheduling permitted. All study personnel had a background in (clinical) psychology or psychiatry and several were members of the Swiss Medical Society of Psycholytic Therapy (SÄPT). Facilitators underwent in-house training to prepare for facilitation LSD sessions in the study. They also regularly visited external training seminars (e.g. via the SÄPT), as well as participating in supervision and intervision groups.

## Dosing session procedures

At least one, and most often two facilitators were always present with a single participant during dosing sessions. Before LSD was administered, facilitators reviewed the plan for the day with participants and asked about any recent stressors that could impact the experience. They then revisited discussions about participants’ readiness to take LSD, strategies for navigating the experience, intentions or wishes, and any remaining questions. A 10-minute guided meditation was offered immediately after drug ingestion with a focus on using the breath as an anchor during the experience, as well as slow breathing to induce relaxation and brief reminders for what to expect from an LSD experience. For the first five hours after dosing, no experiments or activities were planned and participants could freely choose to sit, lie down, or move about the room. They were encouraged to introspect and listen to music, but they could also request silence, talk with the investigators, and do other calm activities (e.g. drawing, reading, light movement). Facilitators usually sat in chairs 1-2 meters away from the participant but asked for participants’ preferences on this and adjusted as needed. No other study staff were present in the room or interacted with participants. Facilitators played music from a pre-programmed playlist chosen to facilitate relaxation and positive mood, unless participants requested otherwise. Participants were asked to bring their own food and drink and were allowed to eat or drink at any time outside of experiments. Facilitators offered light snacks (e.g. fruits), water, and caffeine-free herbal or fruit tea throughout the day.

We used a non-directive guiding approach unless participants requested direction or intervention was needed to ensure safety. Facilitators used various techniques to reduce anxiety or other unwanted effects. The goal of interventions was to increase comfort and prevent an overwhelmingly negative experience, particularly one dominated by feelings of strong fear and helplessness [9]. Support methods included, but were not limited to:

- Verbal reassurance
- Reminders of important information from preparatory sessions, e.g. that participants had taken a drug in a safe setting, they will not be left alone, effects will subside by the evening, etc.
- Suggestions for controlling attention, e.g. observing and slowing the breath, focusing directly on intimidating aspects of the experience to facilitate resolution of unpleasant feelings, or “letting go” and relaxing attention as broadly as possible
- Simple guided meditations (e.g. body scan) or breathing exercises (e.g. square breaths) to facilitate mental and physical relaxation
- Hand-holding or a light hand on the shoulder, only with prior and continuous consent
- Addressing potential external sources of discomfort in the individual (e.g. hunger, thirst, temperature) or environment (lights, sounds)
- Any other strategies deemed to be potentially helpful in the moment

Five hours after dosing, participants were asked to participate in experiments, all of which had been previously encountered in a sober state. It was important to balance experimental rigor with ethical treatment of participants in a strongly altered state of consciousness. Participants were advised that if any experiment was too uncomfortable due to LSD effects, it could be delayed or skipped. Additionally, the schedule was planned with 30-minute time buffers between experiments so that a delay beginning one would not necessarily delay the others. The vast majority of participants were comfortable with the experiments and deviations from protocol were rare. Experiments ended 8-9 hours after dosing. Before sending participants home, the study team briefly discussed the LSD experience with them and issued some practical reminders about what to expect that evening and the next morning. Once LSD effects had sufficiently subsided, participants returned home with a friend or relative, typically 9-10 hours after dosing. There were never any time constraints that caused dosing sessions to end prematurely before a participant wished to return home.

## Preparation for LSD effects

Participants attended two semi-structured preparatory sessions during the first two study visits, each lasting 1-2 hours. The study lead (AEC) was always present and the second facilitator for that participant was present for at least one preparatory session. Because the study was conducted in healthy subjects, we had no therapeutic goals or orientation.

In general, the LSD experience was framed as a journey through an altered state of consciousness in which aspects of one’s mind and environment become strongly amplified or altered. We emphasized that LSD experiences could be interesting or insightful, but sometimes also unpredictable or random. Participants were informed about possible effects on perception, thoughts, emotions, memories, and self-perception, as well as possible adverse effects, using language that strove to be neutral but informative and reflected the most recent scientific understanding. Participants were also advised that LSD sometimes (but not always) has long-term effects on well-being, mood, and outlook on life, which are usually positive but could include rare adverse effects like perceptual changes, mood disturbances, and others [7]; participants were guaranteed ongoing support from the study team should they experience prolonged adverse effects. Additionally, we asked participants about motivations for taking LSD and previous knowledge of LSD. When necessary, we addressed any unrealistic expectations. Participants were encouraged to be as open as possible about questions, concerns, and previous experiences with altered states of consciousness.

The second preparatory session was used to clarify practical aspects (e.g. schedule, what to bring). The role of the facilitators and what to expect from the laboratory setting were discussed in detail, and we asked participants for their preferences about activities, music, interpersonal support, and related topics during the dosing session. Participants were advised that some people like to set a goal or intention before taking LSD to direct the experience, but this was not required. We discussed personal topics which might arise spontaneously during the LSD session, as well as how participants might choose to respond to these. Additionally, we discussed the possibility of experiencing unpleasant or overwhelming effects, particularly fear of losing control. Facilitators explained how strong emotions can sometimes become amplified and how to manage overwhelming feelings (see support methods above), as well as the importance of asking us for help when needed. We emphasized cultivating participants’ trust in their own ability to navigate the experience, but also in the readiness of the study team to assist and accept any reaction to LSD that might manifest.

## Integration protocol

At each of the two follow-up appointments, facilitators conducted a semi-structured integration session lasting 30-90 minutes, depending on participants’ desire to discuss the experience. We discussed the events of the dosing day in roughly chronological order and encouraged participants to freely tell their narrative of the experience while listening in a non-judgmental manner. We inquired about how the evening had gone after returning home, how much participants had slept, and how they were currently feeling the day after LSD. We also specifically asked whether any drug effects that had been particularly surprising, challenging, or positive. We revisited any intentions and discussed possible takeaways from the experience, as well as anything participants wanted support with managing or understanding. Participants were advised that activities like journaling, making art, and discussing the experience with supportive others could help them further process and understand what to make of the experience. They were also reminded that they could request additional support from the study team if they had difficulty processing the experience.

# Supplemental References

1 Batsikadze G, Paulus W, Kuo MF, Nitsche MA. Effect of serotonin on paired associative stimulation-induced plasticity in the human motor cortex. Neuropsychopharmacology. 2013;38(11):2260-7.

2 Rohatgi A. (2024).

3 First MB, Williams JB, Karg RS, Spitzer RL. User's guide for the SCID-5-CV Structured Clinical Interview for DSM-5® disorders: Clinical version. American Psychiatric Publishing, Inc. 2016.

4 Hutten N, Mason NL, Dolder PC, Theunissen EL, Holze F, Liechti ME, et al. Low Doses of LSD Acutely Increase BDNF Blood Plasma Levels in Healthy Volunteers. ACS Pharmacol Transl Sci. 2021;4(2):461-66.

5 Calder AE, Rausch B, Liechti ME, Holze F, Hasler G. Naturalistic psychedelic therapy: The role of relaxation and subjective drug effects in antidepressant response. J Psychopharmacol. 2024;38(10):873-86.

6 Skosnik PD, Sloshower J, Safi-Aghdam H, Pathania S, Syed S, Pittman B, et al. Sub-acute effects of psilocybin on EEG correlates of neural plasticity in major depression: Relationship to symptoms. J Psychopharmacol. 2023;37(7):687-97.

7 Calder AE, Hasler G. Validation of the Swiss Psychedelic Side Effects Inventory: Standardized assessment of adverse effects in studies of psychedelics and MDMA. J Affect Disord. 2024;365:258-64.

8 Pronovost-Morgan C, Greenway KT, Roseman L, Re SE. An international Delphi consensus for reporting of setting in psychedelic clinical trials. Nat Med. 2025;31(7):2186-95.

9 Calder AE, Diehl VJ, Hasler G. Traumatic Psychedelic Experiences. Curr Top Behav Neurosci. 2025:1-26.
